# Light-Activated Virtual Sensor Array with Machine Learning for Non-Invasive Diagnosis of Coronary Heart Disease

**DOI:** 10.1007/s40820-024-01481-7

**Published:** 2024-08-16

**Authors:** Jiawang Hu, Hao Qian, Sanyang Han, Ping Zhang, Yuan Lu

**Affiliations:** 1 Department of Chemical Engineering, Tsinghua University, Beijing, 100084 People’s Republic of China; 2 Key Laboratory of Industrial Biocatalysis, Ministry of Education, Tsinghua University, Beijing, 100084 People’s Republic of China; 3 Department of Cardiology, Xuanwu Hospital, Capital Medical University, Beijing, 100053 People’s Republic of China; 4 Department of Cardiology, Beijing Tsinghua Changgung Hospital, School of Clinical Medicine, Tsinghua University, Beijing, 102218 People’s Republic of China; 5 Institute of Biopharmaceutical and Health Engineering, Shenzhen International Graduate School, Tsinghua University, Shenzhen, 518055 People’s Republic of China

**Keywords:** Black phosphorus/MXene heterostructures, Light-activated virtual sensor array, Diagnosis of coronary heart disease, Machine learning

## Abstract

Early non-invasive diagnosis of coronary heart disease (CHD) is critical. However, it is challenging to achieve accurate CHD diagnosis via detecting breath. In this work, heterostructured complexes of black phosphorus (BP) and two-dimensional carbide and nitride (MXene) with high gas sensitivity and photo responsiveness were formulated using a self-assembly strategy. A light-activated virtual sensor array (LAVSA) based on BP/Ti_3_C_2_T_x_ was prepared under photomodulation and further assembled into an instant gas sensing platform (IGSP). In addition, a machine learning (ML) algorithm was introduced to help the IGSP detect and recognize the signals of breath samples to diagnose CHD. Due to the synergistic effect of BP and Ti_3_C_2_T_x_ as well as photo excitation, the synthesized heterostructured complexes exhibited higher performance than pristine Ti_3_C_2_T_x_, with a response value 26% higher than that of pristine Ti_3_C_2_T_x_. In addition, with the help of a pattern recognition algorithm, LAVSA successfully detected and identified 15 odor molecules affiliated with alcohols, ketones, aldehydes, esters, and acids. Meanwhile, with the assistance of ML, the IGSP achieved 69.2% accuracy in detecting the breath odor of 45 volunteers from healthy people and CHD patients. In conclusion, an immediate, low-cost, and accurate prototype was designed and fabricated for the noninvasive diagnosis of CHD, which provided a generalized solution for diagnosing other diseases and other more complex application scenarios.

**Supplementary Information:**

The online version contains supplementary material available at 10.1007/s40820-024-01481-7.

## Highlights

- Photoresponsive black phosphorus (BP)/Ti_3_C_2_T_x_ composites were synthesized by a self-assembly strategy.
- Enhanced gas sensitive property was achieved by visible light modulation.
- Light activated virtual sensor array was fabricated based on BP/Ti_3_C_2_T_x_ composite.
- Diagnosis of coronary heart disease was achieved with the help of machine learning.

**Supplementary Information:**

The online version contains supplementary material available at 10.1007/s40820-024-01481-7.

## Graphical abstract

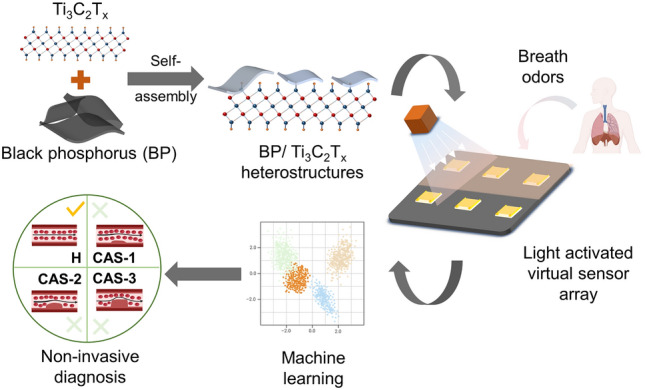

## Introduction

Coronary heart disease (CHD) is one of the most important causes of death worldwide, causing millions of direct deaths each year and numerous sequelae such as disability, cardiac enlargement, and cardiac arrhythmias, so early diagnosis and prevention of CHD is of great significance [1–3]. Diagnostic modalities for CHD such as cardiac stress test, ambulatory electrocardiogram, and coronary computed tomography (CT) angiography, although playing an essential role in the clinic, often require the use of major instruments or complex operations, which hinders the timely detection and treatment of CHD. Therefore, there is an urgent need for a simple, rapid, and accurate testing modality [4–6]. Some studies have reported that human breath has the potential to serve as a new diagnostic source because it contains specific disease markers. For example, the correlation between the concentration of respiratory ammonia and blood urea was explored, and some researchers studied the correlation between exhaled isoprene and blood cholesterol [7, 8]. Regarding CHD, ischemic-damaged cardiomyocytes produce unique biomarkers that will be released through respiration [9]. This demonstrates that exhaled gas is a highly promising candidate for performing noninvasive diagnosis of CHD.

Current gas sensing technologies often rely on large specialized equipment such as tunable diode laser absorption spectroscopy (TDLAS), non-dispersive infrared (NDIR) gas sensors, and ultraviolet-differential optical absorption spectroscopy (UV-DOAS) [10–12]. These technologies with great achievements are widely used in various fields and have an irreplaceable position. However, these devices are not only costly, but also complicated sampling and pre-processing processes make the system response slow, lack portability and ease of operation, and make it difficult to meet the demand for real-time response. Therefore, developing a portable, easy-to-operate, and accurate instant gas sensing platform (IGSP) is significant for the non-invasive diagnosis of CHD patients. The IGSP consists of three parts: the gas sensing module, the signal transmission module, and the data storage and processing module [13]. Among them, the gas sensing module is the most essential part, as its ability to distinguish between different gases determines the accuracy of IGSP. Therefore, it is of great significance to develop an accurate gas sensing module and further build the IGSP based on it.

**Fig. 1:**
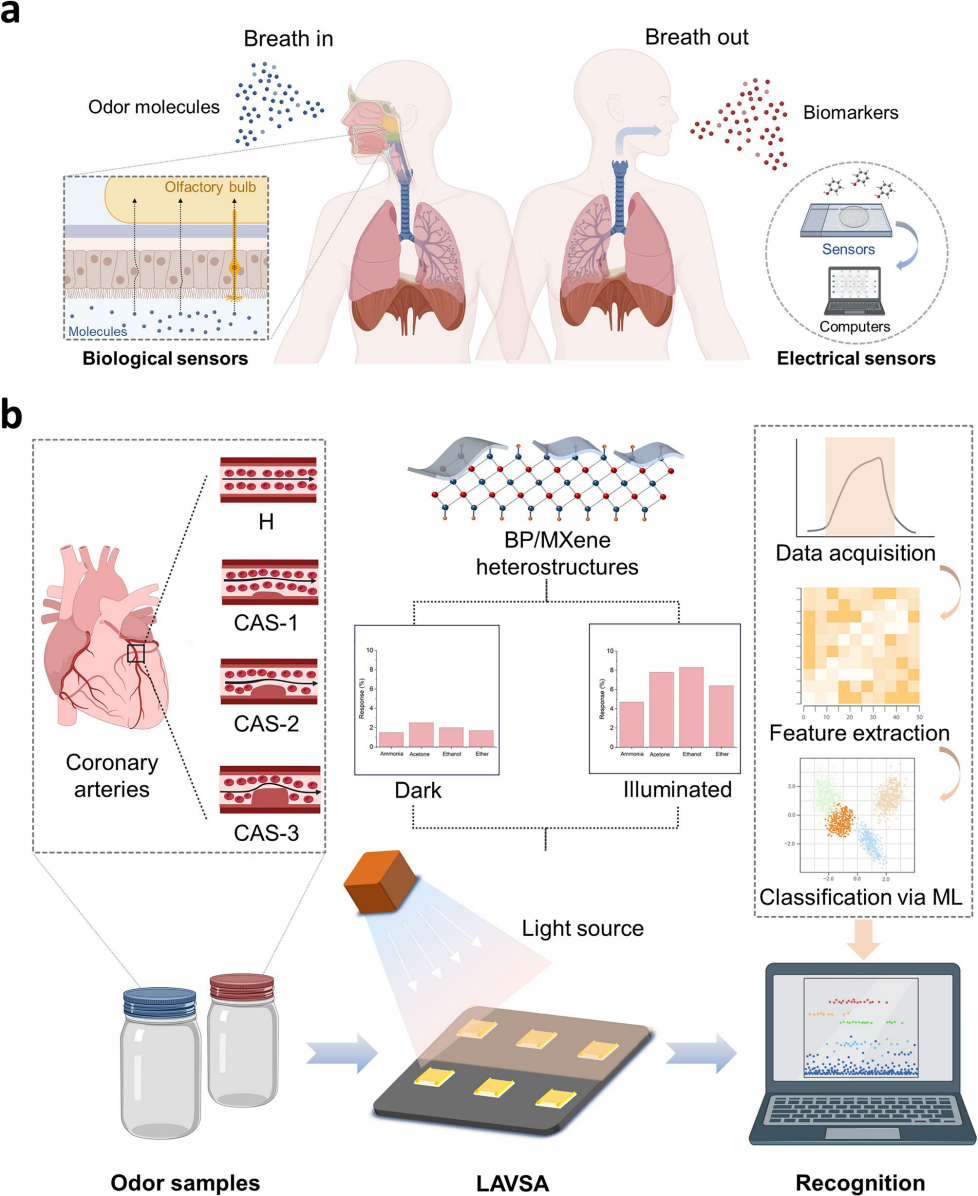
Disease recognition by breath odor through IGSP. **a** Light-activated gas sensing design that mimics the natural olfactory system. **b** Gas detection through IGSP and recognition of different diseases using ML. Figures were created with BioRender

The design aspects of high-performance gas sensing modules have been extensively studied, and researchers have gained inspiration from nature (Fig. 1a). Humans and other mammals can recognize the distinctive odors of volatile chemicals, an ability that stems from the presence of hundreds of subtypes of receptors [14, 15]. Different combinations of receptors can produce unique signals corresponding to various odors. Inspired by this, the sensor array (SA) has been extensively studied [16, 17]. SA consists of multiple sensors of different compositions that are capable of producing unique signals for various odors. For example, one SA for fire detection was prepared using twelve different sensors and achieved 100% accuracy and 85% sensitivity, and another SA consisting of four metal oxide semiconductors, including SnO_2_, In_2_O_3_, WO_3_, and CuO, detected a variety of volatile organic compounds (VOCs) with 98% accuracy [18, 19]. Although the current study is fruitful in the design of SA, the lack of performance of individual sensors and the number of sensors facing diminishing marginal effects need to be addressed.

Sensing materials are the basis for building the SA, with sensitivity and selectivity being key performance indicators. Current commercial chemiresistive gas sensors based on metal oxide semiconductors (MOS: SnO_2_, In_2_O_3_, and CuO et al.) have mature technology and are widely used in industrial processes due to high sensitivity and low cost [20–22]. However, MOS materials face high operating temperatures as well as insufficient selectivity, leading to limitations in their further applications [23, 24]. Other materials such as conductive polymers and perovskites face poor stability and insufficient sensitivity, which are still a long way from practical application [25, 26]. For the performance enhancement of sensing materials, some researchers have explored the potential of novel materials for gas sensing. MXene, a novel two-dimensional (2D) material, is considered to have great potential in gas sensing due to its unique layered structure, excellent electrical conductivity, and abundant terminal groups [27]. For example, a Ti_3_C_2_T_x_ MXene based gas sensors owned good gas-sensing performance with detection limits as low as 50 ppb for VOCs [28]. However, the pristine MXene is similar to other 2D materials and suffers from insufficient selectivity and stability. The good thing is that the gas-sensitive performance has much room for improvement due to its abundant terminal groups [29, 30]. To enhance the gas-sensing performance of MXene-based sensors, chemical modification of MXene nanosheets with second-phase materials is a widely investigated approach. The sensing performance of pristine MXene was improved by changing its structural and electrical properties. In addition, due to the increase in the specific surface area of the composite material, it leads to an increase in the number of active sites for adsorbed gas molecules, which enables better sensitivity to be obtained [31, 32]. For example, in-situ growth of MAPbBr_3_ perovskite on the surface of Ti_3_C_2_T_x_ resulted in a 37%–70% increase in response, and the response speed of W_18_O_49_/Ti_3_C_2_T_x_ composite material was enhanced to 5.6 s [33, 34]. After chemical modification, the stability, sensitivity, and response time were improved to different degrees, which proved that the strategy was effective. However, the choice of the second phase determines the upper limit of the performance enhancement of the composites formed with MXene.

Conventional studies have mainly focused on material replacement or chemical modification, but the degree of improvement usually depends on the gas-sensitive properties of the modifier, which imposes limitations on practical use. In fact, physical environment modification, such as light modulation, is a novel idea of modulation. Changes in the physical environment, such as light, can have an effect on the physicochemical properties of the material, such as faster electron transfer and faster adsorption–desorption processes [35–37]. For example, the response of MoS_2_–NO_2_ was enhanced by 25% using visible light modulation [38]. Inspired by the photosensitizing effect of MXene materials in the range of near-infrared light to visible light, external physical modulation can be utilized to improve the performance of gas sensors based on MXene materials [39, 40]. The introduction of light illumination may result in intrinsic absorption of photons and accelerate the electron transfer between the material surface and the gas molecules. In addition, as other photosensitive materials including MOS and black phosphorus (BP) can form complexes with MXene, not only the light can promote the electron transfer, but also the synergistic effect between the two substances can promote the gas sensing performance [41]. BP is a 2D photosensitive material, and the absorbed light range overlaps with that of MXene as well as be able to form a heterostructure with MXene. Therefore, BP can be a strong competitor for MXene modifiers and has a synergistic effect with MXene when light is introduced [42, 43]. However, there are few reports on BP/MXene complexes as gas sensing feedstock under light modulation. In addition to the material design, the construction strategy of the SA is also important. In principle, the greater the number of sensors, the more accurate the characterization of the gas. However, it also faces diminishing marginal benefits. Inspired by the light modulation strategy, the light-activated virtual sensor array (LAVSA) is proposed, where the SA is tested for gases with and without light conditions separately. It is equivalent to doubling the number of sensors in the SA, enabling a more accurate portrayal of the target gas. This strategy can geometrically increase the efficiency of SA. However, there are few reports on LAVSA in the field of gas sensing, let alone the IGSP based on it.

In addition to the study of materials, the algorithms used to process the data have a significant impact on the performance of the IGSP. Because of the large amount of data obtained from experiments, accurately extracting features from different samples and classifying them is the crux of the problem. Machine learning (ML) algorithms, a category of Artificial Intelligence, are able to simulate humans to learn and analyze data by monitoring and distinguishing relationships between data. There are many algorithms of ML based on different principles, such as Support Vector Machines (SVMs) and Naive Bayes (NB) [44]. Due to its ability to extract features from large amounts of data, ML has many applications in diagnosing and predicting different diseases. For example, Gupta et al. used NB algorithm to estimate the risk of coronary heart disease with an accuracy of 0.93 [45]. In addition, Joloudari et al. used Decision Tree (DT) algorithm for coronary heart disease diagnosis with an accuracy of 91.47% [46]. However, there are few studies on using ML for gas sensing systems, let alone diagnosing coronary heart disease via gas samples.

Based on the above research ideas and current status, this work aims to prepare LAVSA with high gas-sensing properties by MXene-based photoresponsive composites and further fabricate easy-to-operate and accurate IGSP (Fig. 1b). Further, the prepared IGSP was used for the detection and noninvasive diagnosis of exhaled breath of CHD patients and healthy individuals with the help of the ML algorithm. BP/Ti_3_C_2_T_x_ complexes were first synthesized by self-assembly strategy and prepared as sensors. As a proof-of-concept, detection was performed using typical gases, including ammonia, acetone, ethanol, and ether, to explore the optimal composition, light wavelengths and intensities, and the enhancement of sensing performance of BP/Ti_3_C_2_T_x_. Subsequently, a six-dimensional LAVSA was fabricated based on three materials, i.e., pure Ti_3_C_2_T_x_, pure BP, and BP/Ti_3_C_2_T_x_ composite. Fifteen odor molecules, which are widely present in nature, were tested with and without illumination to examine the gas recognition performance of LAVSA. Based on this, an IGSP was prepared using the LAVSA and Arduino platform, which was applied for the non-invasive diagnosis of CHD patients. A total of 45 breath samples from CHD patients and healthy individuals were examined using IGSP for identification and diagnosis with the help of ML. In a word, this work developed a LAVSA-based IGSP, which provided a new strategy for the construction of the SA and noninvasive diagnosis of CHD, and provides ideas for noninvasive and immediate detection of other diseases such as cancer and gastric diseases. 

## Experimental Section

### Materials

Lithium fluoride (LiF, 99.99%, 100 μm) was supplied by Merck Co., Ltd. Ti_3_AlC_2_ powder (90%, 40 μm) was supplied by Merck Co., Ltd. Hydrochloric acid (HCl, AR) was supplied by Meryer Co., Ltd. N, N-dimethylformamide (DMF, SafeDry) was supplied by Aladdin Co., Ltd. Tetrabutylammonium hydroxide (TBAOH, 40%) was supplied by Bidepharm Co., Ltd. Black phosphorous (AR, 99.9%) nanosheets were purchased from Zhongke Materials Co., Ltd.

### Preparation of Few-Layer Ti_3_C_2_T_x_ Nanosheets

Ti_3_C_2_T_x_ nanosheets dispersed in DMF were prepared using the tuned microenvironment method. In a polytetrafluoroethylene (PTFE) reactor, 4 g of LiF was added to 80 mL of 9 M HCl solution and stirred at 35 °C for 60 min to dissolve. Then, 4 g of Ti_3_AlC_2_ was added and stirred at 35 °C and 1000 rpm for 24 h. Finally, the collected multilayer Ti_3_C_2_T_x_ MXene is freeze-dried for 36 h. Then, 1 g of multilayer Ti_3_C_2_T_x_ powder was added to 24 mL of 25% TBAOH solution. The resulting product was stirred at 500 rpm for 6 h at room temperature for intercalation. Excess TBAOH was then washed off with ethanol, and all precipitate was collected in a 100 mL centrifuge tube. Approximately 60 mL of DMF was then added to the centrifuge tube and shaken until the precipitate completely disappeared. The upper liquid layer was collected by centrifugation at 12,000 rpm for 5 min. DMF was added and centrifuged repeatedly until a 50 mL solution was collected with a concentration of approximately 5 mg mL^−1^ of MXene.

### Preparation of BP/Ti_3_C_2_T_x_ Composite

To prepare BP/Ti_3_C_2_T_x_ composite, 20 mL of MXene DMF (~ 5 mg mL^−1^) solution was added to the reactor, and then 0.1 g of BP nanosheets was added for the self-assembly process. The mixture was shaken for 30 s to obtain a targeted homogeneous black solution. A portion of the solution was dried to obtain solids, and the composite was denoted as BP/Ti_3_C_2_T_x_.

### Preparation of Light-Activated Virtual Sensor Array (LAVSA)

0.5 mL solutions of the Ti_3_C_2_T_x_, BP, and BP/Ti_3_C_2_T_x_ were added to 10 mL of DMF, respectively, to obtain three solutions as the primary sources for the gas-sensitive films. Then, three interdigital electrodes served as the backbone of LAVSA. A spray gun was utilized to evenly apply 2 mL of each solution of the three synthesized materials to the interdigital electrode, and then electrodes were dried in a vacuum oven at 60 °C for 2 h to form a gas-sensitive film. The LAVSA was used in the presence and absence of light to obtain six-dimensional data. Each sensor of the obtained MBA was recorded as Sensor 1: (without light) BP/Ti_3_C_2_T_x_, Sensor 2: (under light) BP/Ti_3_C_2_T_x_. Sensor 3: (without light) Ti_3_C_2_T_x_, Sensor 4: (under light) Ti_3_C_2_T_x_, Sensor 5: (without light) BP, and Sensor 6: (under light) BP.

### Structural Characterizations

X-ray diffraction (XRD, MiniFlex600, Rigaku, Japan) was performed using Cu Kα radiation (λ = 0.15406 nm) at a voltage of 40 kV, a current of 15 mA, a scanning frequency of 0.083° s^−1^, and a scanning range of 3°–60° in the 2θ region to explore crystal properties. The surface morphology was characterized using a field emission scanning electron microscope (FESEM: SU8220, HITACHI, Japan) equipped with an energy-dispersive X-ray spectrometer (EDS) with an accelerating voltage of 10 kV. The shape and morphology were analyzed with a high-resolution transmission electron microscope (HRTEM: JEM2010, JEOL, Japan) to observe the lattice of Ti_3_C_2_T_x_. X-ray photoelectron spectroscopy (XPS) analysis was performed on an X-ray photoelectron spectrometer (AXIS Supra + , Kratos, UK) using an Al Kα X-ray source to investigate the surface electronic states. The morphology and thickness of the samples on silicon substrates were studied using an atomic force microscope (AFM: Dimension ICON, Bruker, Germany) in ScanASYST mode. Gas chromatography-mass spectrometry (QP2010 ultra, Shimadzu, Japan) was used to identify the components in odor samples from different volunteers.

### Dynamic Gas Sensing Experiment

Gas sensing measurements were made at room temperature using a self-built Instant gas sensing platform (IGSP). A 1.2-L vessel was used as a vapor-sensing chamber. During the experiment, different volumes of the gas solution to be tested were directly fed into the vessel through a hole located on the upper surface of the vessel. After injection, the vessel was sealed with transparent tape. The concentration of the test gas was determined by the following calculation:

$$C \left( {ppm} \right) = \frac{\rho \cdot V \cdot \omega }{M} \cdot \frac{{V_{m} }}{{V_{c} }} \times 10^{6}$$

where $\rho$ (g mL^−1^) is the density of the gas molecule; $V \left( {\mu L} \right)$ is the volume of the gas molecule solution; $\omega { }\left( \% \right)$ is the mass fraction of the gas molecule solution; *M* (g mol^−1^) is the molar mass of the gas molecule; $V_{m}$ (L mol^−1^) is the molar volume of the ideal gas; $V_{c} \left( L \right)$ is the volume of the vapor sensing chamber.

Resistance values were recorded under steady-state conditions after exposure to pure air and air containing the target gas using an Arduino-based gas sensing system. The Arduino board facilitated data acquisition and storage for dynamic gas detection. An advanced computer program collected data from all sensors at a constant rate. The applied DC voltage had an amplitude of 5 V and a measurement error of less than 0.1%.

### Identification of CHD Patients via Breath Odors

The IGSP was applied to detect and identify respiratory gases in different populations. In all, 45 volunteers took part in this study, including 10 healthy individuals (H), 11 patients with degree of CAS 0–25% (CAS-1), 9 patients with degree of CAS 25%–50% (CAS-2), and 15 patients with degree of CAS more than 50% (CAS-3). Before the study, informed written consent was obtained from all participants. The approval from the institutional ethics committee, Biomedical Research Ethics Committee, Department of Cardiology, Beijing Tsinghua Changgung Hospital was obtained prior to the research (approval number: 24034–4-01).

Before the experiment, well-sealed aluminum gas collecting bags with a volume of 1 L were used to collect the exhaled gas from volunteers. The outlet of the bag then delivered the gas smoothly to the LAVSA over a distance of 2 cm for 10 s. The computer recorded the change in LAVSA resistance in real-time. To ensure the stability of the experiment, 20 s were spaced between each delivery to restore the baseline resistance of the LAVSA. A total of 20 consecutive experiments in the presence and absence were performed on breath samples from each volunteer.

## Results and Discussion

### Fabrication and Characterization of BP/Ti_3_C_2_T_x_-Based Composite and Gas Sensor

**Fig. 2:**
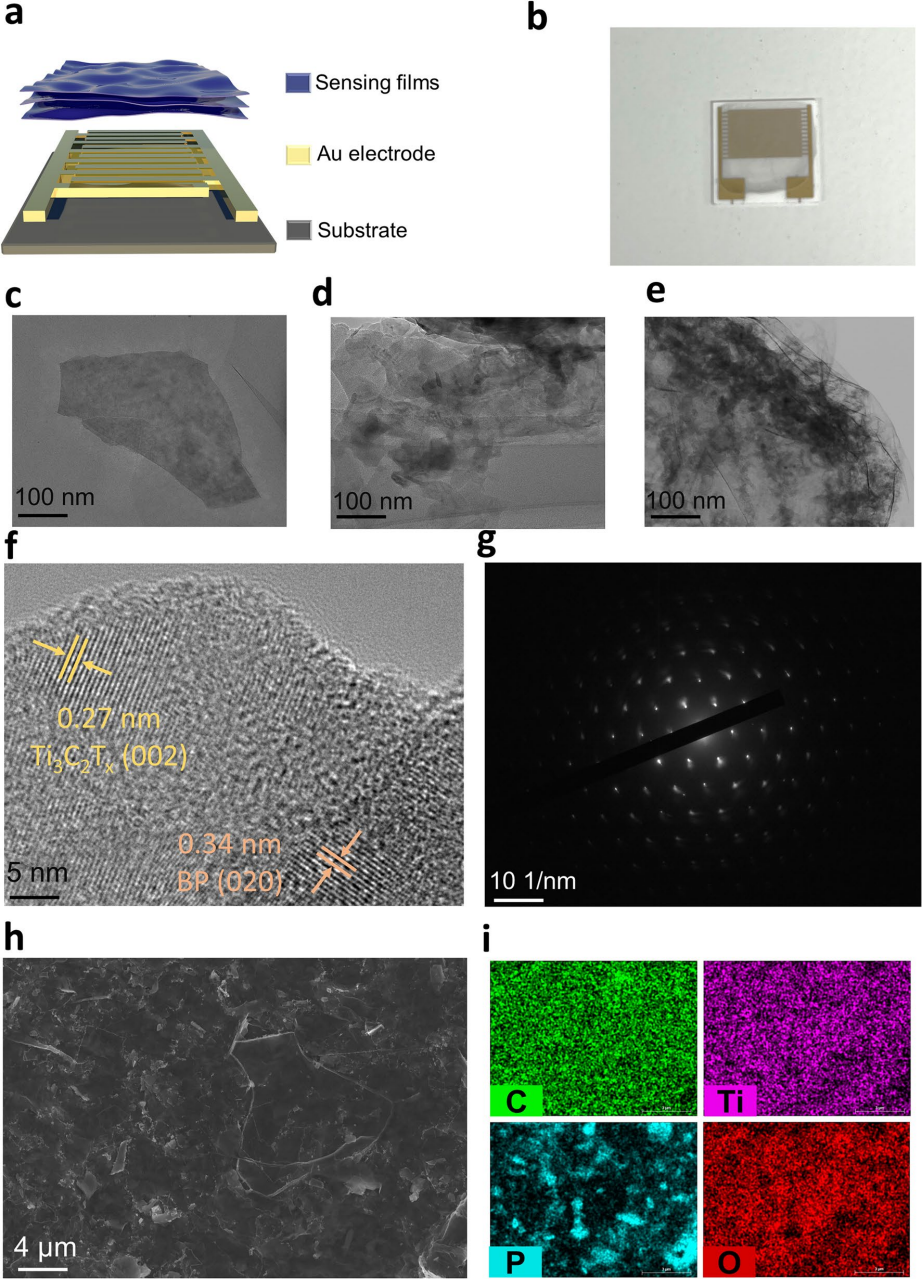
Physical, micro, and crystal structure of the gas sensor. **a** Diagram of the physical structure of the gas sensor. **b** Real picture of the gas sensor. TEM Images of **c** Ti_3_C_2_T_x_ nanosheets, **d** BP nanosheets and **e** BP/Ti_3_C_2_T_x_ composite. **f** Image of HRTEM of BP/Ti_3_C_2_T_x_ composite. **g** Image of SAED pattern of BP/Ti_3_C_2_T_x_ composite. **h** Image of SEM of the surface of BP/Ti_3_C_2_T_x_ composite. **i** Image of EDS elemental mapping of BP/Ti_3_C_2_T_x_ composite

The core components of a gas sensor were sensing materials with different properties. MXene nanosheets were prepared using the tuned microenvironment method (Fig. S1) [47]. Due to the ease of tunability of MXene surface groups, forming composites with different materials was a means to significantly improve gas sensing performance. As shown in Fig. S2, BP/Ti_3_C_2_T_x_ composites were prepared by a self-assembly process, and their surface morphology, physical and chemical structures were characterized. The Ti_3_C_2_T_x_, BP, and BP/Ti_3_C_2_T_x_ solutions were shown in Fig. S3. As shown in Fig. 2a, the gas sensor consisted of a substrate, an electrode, and a gas-sensitive membrane. The specific preparation process was to fix an interdigital electrode on the polyethylene terephthalate (PET) substrate as the backbone of the gas sensor, and then the synthesized composite was uniformly sprayed on the interdigital electrode to form a gas-sensitive membrane. The fabricated gas sensor was shown in Fig. 2b.

To study the surface morphology of the material, the samples were observed using scanning electron microscopy (SEM), transmission electron microscopy (TEM), and atomic force microscopy (AFM). The morphology of Ti_3_C_2_T_x_ nanosheets was shown in TEM images (Fig. 2c) as uniform thin layers, indicating a typical two-dimensional structure. The thickness of Ti_3_C_2_T_x_ nanosheets was shown to be around 3 nm in the AFM images (Fig. S5a, b), which was consistent with the TEM results [48]. The images of TEM of BP also showed a stacked sheet structure (Fig. 2d), indicating the two-dimensional material properties of BP. The thickness of BP nanosheets was shown to be around 30 nm in the AFM images and was uniform in thickness (Fig. S5c, d) [49]. The TEM images of the heterostructures of BP/Ti_3_C_2_T_x_ showed the self-assembly of BP nanosheets on the surface of Ti_3_C_2_T_x_ nanosheets (Fig. 2e). To further investigate the BP/Ti_3_C_2_T_x_ heterostructure, high-resolution transmission electron microscopy (HRTEM) was employed to observe the crystal properties. The HRTEM images (Fig. 2f) of the BP/Ti_3_C_2_T_x_ heterostructure showed that there were two distinctly different widths of lattice spacings of 0.27 and 0.34 nm, which corresponded to the (002) plane of the Ti_3_C_2_T_x_ and the (020) plane of BP [43, 50, 51]. The corresponding selected area electron diffraction (SAED) patterns (Fig. 2g) showed bright spots consistent with the lattice spacing, indicating that the BP/Ti_3_C_2_T_x_ heterostructures exhibit typical crystalline properties. The elemental distribution and film-forming morphology of the samples were examined by SEM and energy dispersive spectroscopy (EDS). SEM images of pure Ti_3_C_2_T_x_ (Fig. S4b) showed the formation of a uniform film, indicating the successful preparation of Ti_3_C_2_T_x_ nanosheets, and the uniform distribution of Ti, O, and C elements can be seen by EDS elemental mapping. The SEM images of BP showed the successful preparation of BP nanosheets, and the uniform distribution of P could be seen by EDS elemental mapping (Fig. S4a). The SEM images of the composites demonstrated a stacked membrane-like structure, proving that BP self-assembled on Ti_3_C_2_T_x_ to form a nanocomposite structure. EDS elemental mapping confirmed the distribution of elements C, O, P, and Ti in the BP/Ti_3_C_2_T_x_ nanocomposites (Fig. 2h, i), suggesting that BP self-assembled on Ti_3_C_2_T_x_. In summary, the surface morphology and elemental distribution based on BP, Ti_3_C_2_T_x_, and BP/Ti_3_C_2_T_x_ indicated that BP was successfully self-assembled onto Ti_3_C_2_T_x_ and formed a heterogeneous structure.

**Fig. 3:**
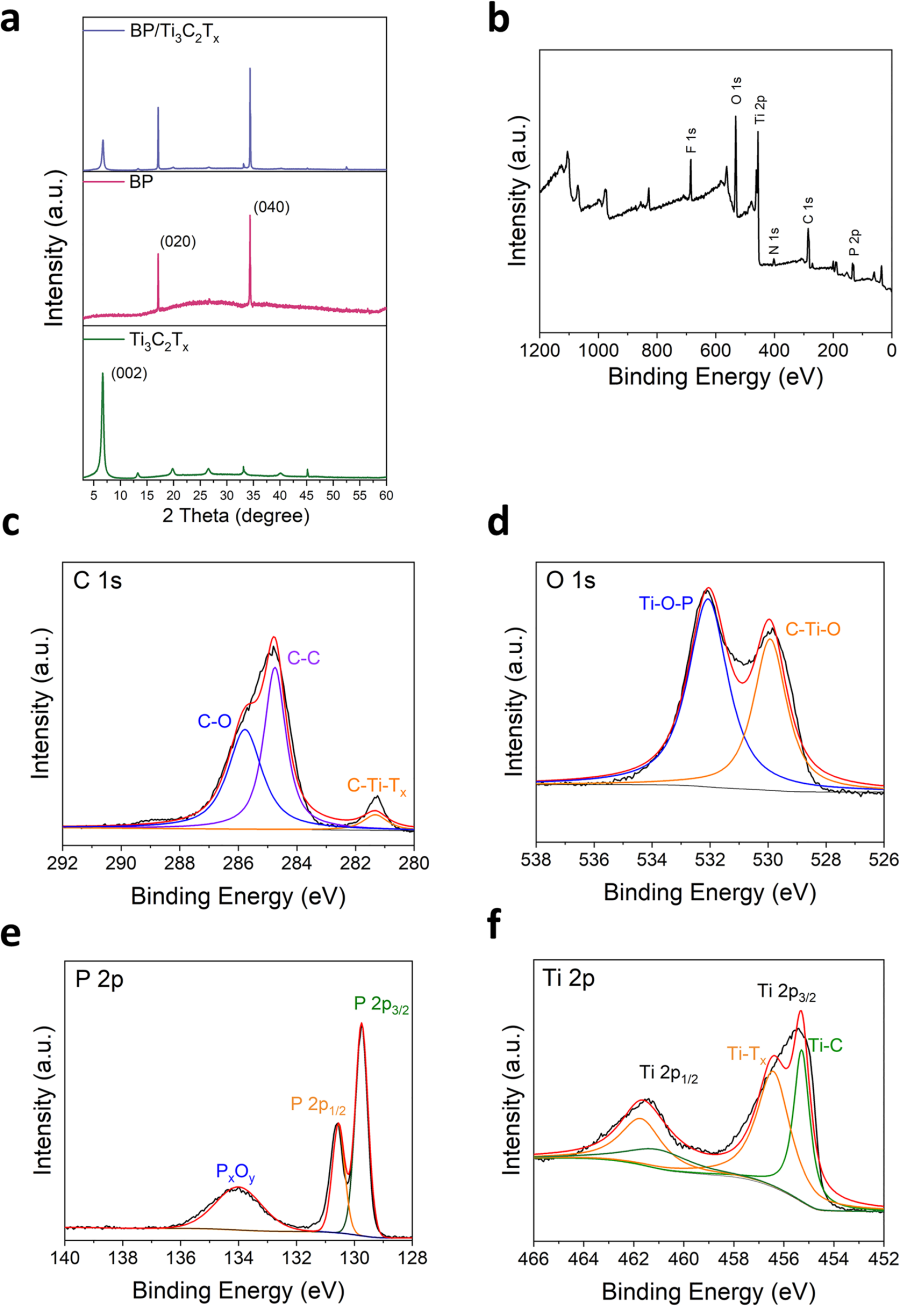
Characterization of crystal and chemical properties. **a** Image of XRD of BP, Ti_3_C_2_T_x_, and BP/Ti_3_C_2_T_x_ composite. **b** XPS survey spectrum of BP/Ti_3_C_2_T_x_ composite and high-resolution XPS spectra of **c** C 1*s*, **d** O 1*s*, **e** P 2*p* and **f** Ti 2*p* in BP/Ti_3_C_2_T_x_ composite, respectively

The synthesized BP/Ti_3_C_2_T_x_ composites, Ti_3_C_2_T_x,_ and BP were analyzed by XRD to determine the crystal structure and phase composition. The characteristic diffraction peaks of BP were located at 2θ = 17.5° and 34.9° corresponding to (020) and (040) crystal planes, respectively [52, 53]. Ti_3_C_2_T_x_ had a distinct cusp at about 7.1° corresponding to the (002) crystal plane [54, 55]. The heterostructure of BP/Ti_3_C_2_T_x_ was mainly confirmed by observing whether the diffraction peaks of BP and Ti_3_C_2_T_x_ nanosheets were present at the same time or not. As BP self-assembled onto the surface of Ti_3_C_2_T_x_, the peak intensity of Ti_3_C_2_T_x_ gradually decreased (Fig. 3a). This was due to the fact that X-rays needed to penetrate the BP nanosheets in order to reach the Ti_3_C_2_T_x_ layer underneath, which led to a decrease in the peak intensity of Ti_3_C_2_T_x_. The simultaneous appearance of the characteristic peaks of BP and Ti_3_C_2_T_x_ in the XRD spectra proved the successful formation of the BP/Ti_3_C_2_T_x_ heterostructures with a good crystal structure.

XPS analysis was performed to investigate the surface chemical composition and bonding state of the BP/Ti_3_C_2_T_x_ composites. The wide spectrum (Fig. 3b) showed the presence of Ti, C, P, O, F, and N elements in the BP/Ti_3_C_2_T_x_ nanocomposites. The high-resolution C 1*s* spectrum (Fig. 3c) showed three components at 281.21, 284.53, and 286.47 eV, corresponding to C–Ti–O, C–C, and C–O, respectively [56, 57]. The high-resolution O 1*s* spectrum (Fig. 3d) showed two strong peaks at 529.95 and 532.27 eV, corresponding to C–Ti–O and Ti–O–P, respectively [56–58]. The high-resolution P 2*p* spectrum (Fig. 3e) observed strong peaks at 129.72 and 130.83 eV corresponding to P 2*p*_3/2_ and P 2*p*_1/2_, respectively, as well as at 134.12 eV corresponding to P_x_O_y_, confirming BP self-assembled in Ti_3_C_2_T_x_ [52, 59]. High-resolution Ti 2*p* spectrum (Fig. 3f) showed two double peaks with an area ratio of 1:2, i.e., Ti 2*p*_1/2_ and Ti 2*p*_3/2_. Two strong peaks of Ti 2*p*_3/2_ were observed at 455.32 and 456.51 eV, corresponding to Ti–C and Ti–T_x_, respectively [55, 60, 61]. It suggested that in Ti_3_C_2_T_x_, Ti–C, and Ti–T_x_ bonds were the dominant chemical state of Ti, and there was no material oxidation. In conclusion, the XPS analysis revealed important information about the chemical composition and bonding states for the BP/Ti_3_C_2_T_x_ heterostructure, providing evidence for the successful preparation of the complex.

### Sensing Properties of BP/Ti_3_C_2_T_x_ Composite

**Fig. 4:**
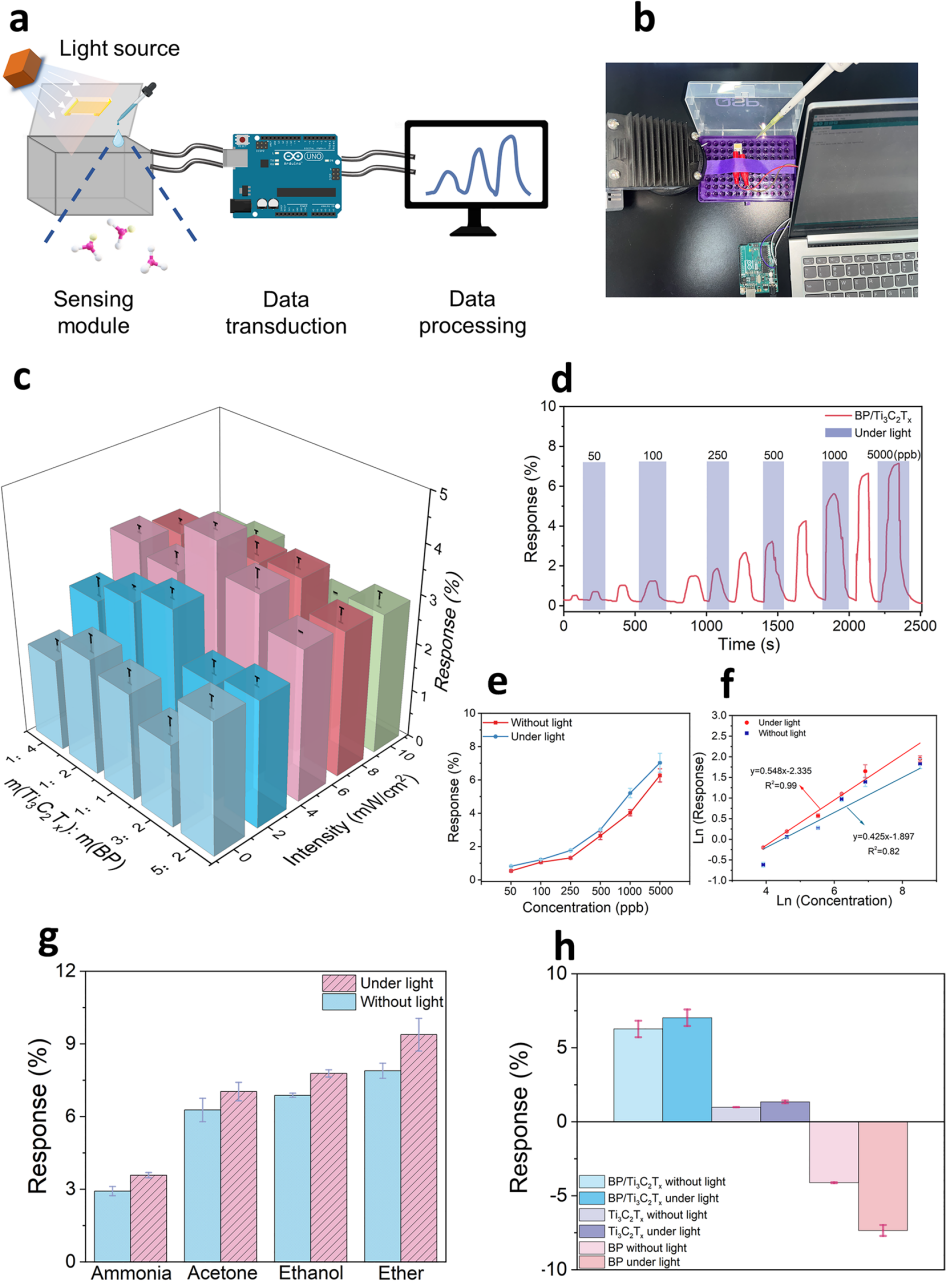
Structure of IGSP and sensing performance of the BP/Ti_3_C_2_T_x_ composite. **a** Diagram of the working of LAVSA-based IGSP. Figures were created with BioRender. **b** Real picture of the sensing module. **c** Response of different ratios of BP/Ti_3_C_2_T_x_ composite at different light intensities at 420 nm to acetone at the concentration of 5 ppm. **d** Sensing performance of BP/Ti_3_C_2_T_x_ composite to acetone at concentrations from 50 ppb to 5 ppm in the presence and absence of light. **e** Comparison of the performance of the BP/Ti_3_C_2_T_x_ composite in the presence and absence of light to acetone at concentrations ranging from 50 ppb to 5 ppm. **f** Comparison of the logarithm linear relationship between the response and the acetone concentration of BP/Ti_3_C_2_T_x_ composite in the presence and absence of light. **g** Comparison of the performance of the BP/Ti_3_C_2_T_x_ composite in the presence and absence of light to ammonia, acetone, ethanol, and ether of 5 ppm. **h** Comparison of the response of the Ti_3_C_2_T_x_, BP, and BP/Ti_3_C_2_T_x_ composite to acetone at the concentration of 5 ppm in the presence and absence of light

The performance of dynamic response to gas was one of the most important criteria for judging the goodness of a gas sensor. The sensor response was defined as $\frac{{\text{R}}_{\text{g}}-{\text{R}}_{\text{a}}}{{\text{R}}_{\text{a}}}\times 100 {\%}$, where ${\text{R}}_{\text{a}}$ was the initial resistance of the sensor in air, and ${\text{R}}_{\text{g}}$ was the resistance of the sensor when exposed to the target gas. In this work, experiments were designed based on the law of conservation of matter and the principle of gas–liquid equilibrium, and the experimental setup consisted of a sensing module, a data transmission platform, and a data processing module (Fig. 4a). In the experiment, the sensor was placed in a target gas environment with changing concentration in a closed chamber to examine the dynamic response. The gas concentration was varied by dropping different volumes of a solution of gas molecules (Fig. 4b), which utilized the ability of the solution and the gas molecules to form a gas–liquid phase equilibrium [62]. An analog signal was acquired through the sensor, which was then converted into an electrical signal and transmitted to a data processor.

The introduction of light modulation to enhance the gas-sensitive properties of the sensors was a promising way. In this work, BP was self-assembled onto the MXene surface to form BP/Ti_3_C_2_T_x_ composite and prepared as gas sensors. As a validation experiment, different ratios of BP/Ti_3_C_2_T_x_ sensors were synthesized and tested for typical gases such as acetone, ammonia, ether, and ethanol under different wavelengths and intensities of light modulation to determine the optimal ratios as well as the optimal light conditions.

Firstly, the optimal ratio between BP and Ti_3_C_2_T_x_, as well as the optimal light wavelength and intensity, were explored and tested using acetone. Since the light absorption ranges of A and B were between the visible and near-infrared, representative wavelengths of 420 and 800 nm were selected for the study, according to previous reports [63–66]. It could be seen that the sensor response was different at wavelengths of 420 and 800 nm light (Figs. 4c and S6), and the sensor had a higher response at 420 nm. In addition, as the BP content increased, the sensor response had a convex function nature with the highest response at a mass fraction of 1:1. In addition, with the gradual increase in light intensity, the sensor response also had a convex function nature and had the highest response at a light intensity of 5 mW cm^−2^. In summary, the sensor had the optimal response when the illumination was at 420 nm, the intensity was 5 mW cm^−2^ and the mass fraction ratio of BP and Ti_3_C_2_T_x_ was 1:1, and the following experiments were based on this condition.

To compare the effect of light on the response, gases from 50 ppb to 5 ppm were tested by the BP/Ti_3_C_2_T_x_-based sensor in the presence and absence of light, respectively. Figure 4d and Fig. S7a–c showed the real-time resistance of the BP/Ti_3_C_2_T_x_-based sensor to acetone, ammonia, ethanol, and ether. It could be seen that the change in the resistance of BP/Ti_3_C_2_T_x_ in the presence of light was higher than that in the absence of light due to the fact that light promoted the electron exchange of gas molecules on the surface of BP/Ti_3_C_2_T_x_. The resistance of the sensor increased in the target gas environment with or without light. This might be attributed to the electron transfer due to the adsorption of the gas molecules by the surface groups of BP/Ti_3_C_2_T_x_, resulting in a decrease in the concentration of hole carriers inside BP/Ti_3_C_2_T_x_. Notably, at 5 ppm, the response values of the BP/Ti_3_C_2_T_x_-based sensors for acetone, ammonia, ether, and ethanol under light were 7.21%, 3.67%, 9.32%, and 7.55%, respectively, which were 1.19, 1.22, 1.26, and 1.07 times of the responses without light (Figs. 4e and S8a–c). This might be due to the photon excitation of BP/Ti_3_C_2_T_x_ surface electrons, which promoted the adsorption of gas molecules onto the material surface, thus reducing the carrier density in Ti_3_C_2_T_x_ and improving the sensitivity. When the gas concentration increased, the number of carriers decreased, and the channel resistance increased, thus increasing the sensor resistance.

Compared with the reported light-activated gas sensors, the BP/Ti_3_C_2_T_x_ gas sensor responded significantly to the target gas and exhibited excellent gas-sensitive performance (Table S1). In addition, to visualize the relationship between the target gas concentration and the sensor response, the natural logarithm of the response values of the BP/Ti_3_C_2_T_x_-based sensors in the presence and absence of light was linearly fitted to the natural logarithm of the gas concentration. As shown in Figs. 4f and S9a–c, for acetone and ether, the slope in the presence of light was larger than that in the absence of light as the gas concentration increased. While the slope of ammonia and ethanol in the presence of light was less than that in the absence of light. The results showed that the BP/Ti_3_C_2_T_x_ based sensor was more sensitive to the gas of acetone and ether in the presence of light, which might be due to the stronger adsorption capacity of BP/Ti_3_C_2_T_x_ for them in the presence of light. Notably, the BP/Ti_3_C_2_T_x_-based sensor showed good linearity for these gases in the range of 50 ppb to 5 ppm, suggesting that the sensor could be further practically applied for real-time monitoring and concentration measurement where the concentration range spanned 100-fold.

To compare the selectivity of BP/Ti_3_C_2_T_x_ in the presence and absence of light conditions, each of the above four gases was tested at 5 ppm. The results were shown in Fig. 4g, where the difference between the responses of the different gases became larger in the presence of light, indicating that the selectivity of the gases was improved. In addition, to compare the effect of light on the gas sensing performance of Ti_3_C_2_T_x_, BP, and BP/Ti_3_C_2_T_x_ composites, the four gases were tested at 5 ppm with and without light conditions, respectively. The results were shown in Figs. 4h and S10a–c, where the three materials had responses of 1.13%, − 4.03%, and 6.06% without light. In the presence of light, the corresponding responses were 1.07, 1.76, and 1.19 times higher, respectively, indicating that the light made the response differences between the sensors larger thus owning greater selectivity.

In a word, the response of BP/Ti_3_C_2_T_x_ to different gases was enhanced to varying degrees under light, resulting in greater variability in the gas response and thus improved selectivity. In addition, due to the nature of Ti_3_C_2_T_x_ itself and the synergistic effect with BP, its sensitivity remained high. This was important for detecting low concentration target gases in practical application scenarios. Therefore, the high sensitivity of BP/Ti_3_C_2_T_x_ under light made it a strong contender for gas sensors and could be further prepared with BP and Ti_3_C_2_T_x_ to form the LAVSA with excellent performance.

### Sensing Characteristics of LAVSA

Previous studies on gas sensor arrays typically required testing a large number of different materials to obtain a single sensor with unique properties, and the marginal benefit decreased as the number of sensors was raised. Ti_3_C_2_T_x_, BP, and BP/Ti_3_C_2_T_x_ themselves had large differences for gases, and photomodulation could increase this difference. In this work, inspired by the photomodulation strategy, if a SA made of these three materials detected gases in the presence and absence of light, which allowed for two times the gas sensing data, and the dimensionality of the sensors was equivalent to doubling. The original SA consisted of three single sensors, and under light modulation, it was equivalent to introducing three virtual sensors to prepare the LAVSA. This not only simplified the preparation process of the SA, but also maintained the good performance. The LAVSA fabricated in this work consisted of six sensors, including Sensor 1: (without light) BP/Ti_3_C_2_T_x_, Sensor 2: (under light) BP/Ti_3_C_2_T_x_. Sensor 3: (without light) Ti_3_C_2_T_x_, Sensor 4: (under light) Ti_3_C_2_T_x_, Sensor 5: (without light) BP, and Sensor 6: (under light) BP.

**Fig. 5:**
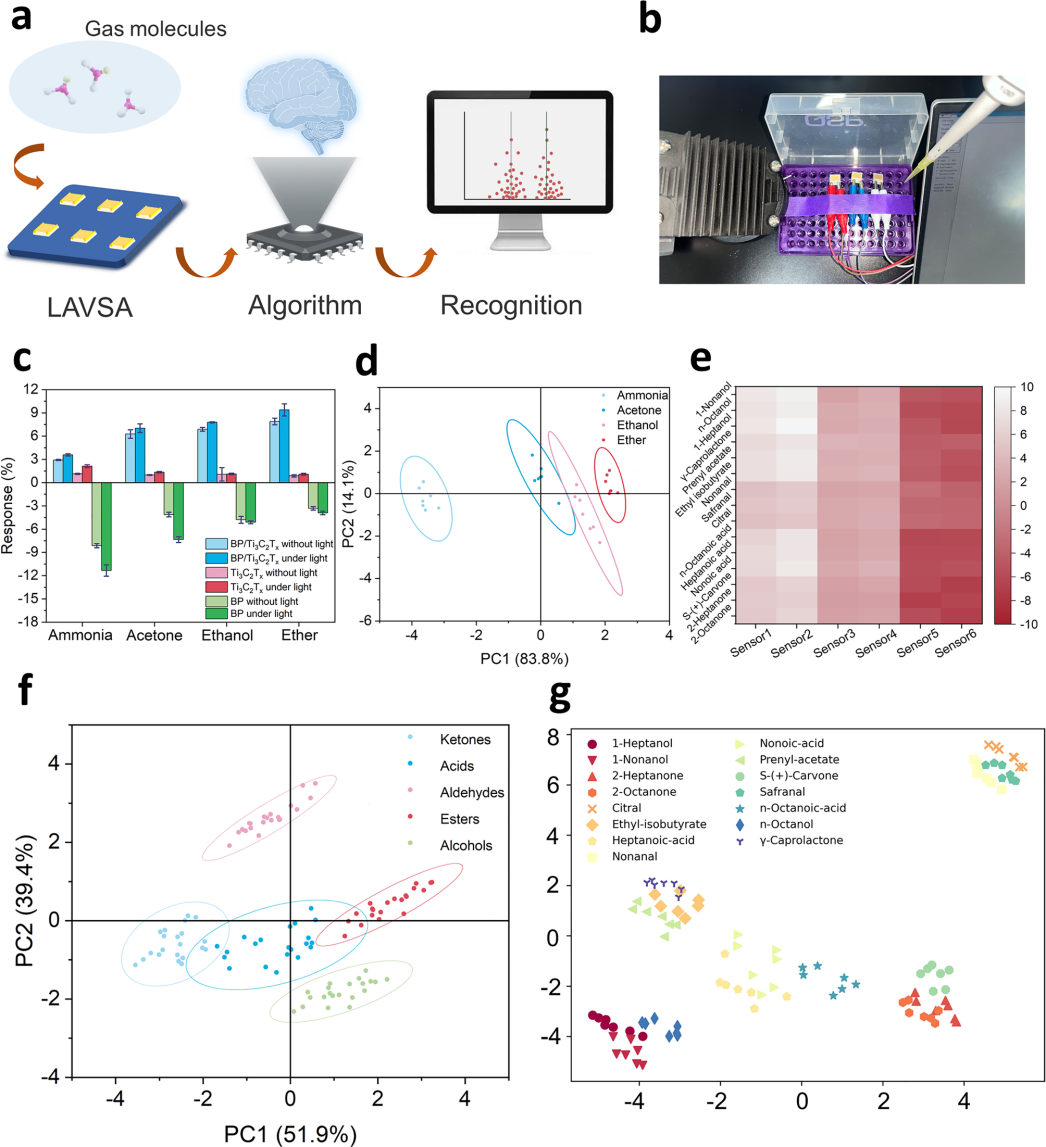
Gas sensing performance of the LAVSA. **a** Diagram of the working of the LAVSA. Figures were created with BioRender. **b** Real picture of the LAVSA. **c** Gas sensing performance of the LAVSA exposed to ammonia, acetone, ethanol and ether of 5 ppm. **d** Image of PCA of the LAVSA to ammonia, acetone, ethanol, and ether of 5 ppm. **e** Average sensing performance of individual sensor in LAVSA to fifteen natural gas molecules of 5 ppm. **f** Image of PCA of the LAVSA to five types of natural gas molecules of 5 ppm. **g** Image of t-SNE of the LAVSA to fifteen natural gas molecules of 5 ppm

First, the four gases mentioned above were detected to test the sensitivity performance of the LAVSA. The sensitivity of the LAVSA was tested by exposing it to a target gas environment ranging from 50 ppb to 5 ppm. After collecting the data, a pattern recognition algorithm was utilized to differentiate and identify the different gases (Fig. 5a, b). As shown in Fig. S11a–d, the response of each sensor was enhanced with increasing target gas concentration. Due to the different compositions of the sensors, they responded to gases to various degrees (Fig. 5c). For example, for 5 ppm ammonia, the response of sensor (with light) BP/Ti_3_C_2_T_x_ was 3.67%, while sensor (with light) BP was − 11.03%. This difference could be used to demonstrate the selectivity of the LAVSA. Seven experiments were conducted for each of the four gases mentioned above at a concentration of 5 ppm, and the data were analyzed using principal component analysis (PCA) [67]. Figure 5d shows that the various gases clustered in a corresponding region, indicating that the LAVSA was able to differentiate between these gases at a concentration of 5 ppm, and therefore had excellent selectivity for these gases. This was mainly attributed to the selective accumulation of the six sensors in the LAVSA, which maximized the differentiation of the various gases by characterizing them in six dimensions. This indicated the successful fabrication of the LAVSA and demonstrated that it was constructed from individual sensors prepared with different ratios of BP to Ti_3_C_2_T_x_. It had remarkable results in selectively recognizing the target gases under light modulation.

To test the actual performance of the LAVSA, 15 odor molecules (Table S2) were introduced and tested accordingly, choosing a total of five categories of substances, i.e., alcohols, ketones, aldehydes, esters, and acids. These molecules were widely found in nature and were therefore typical (Fig. S13). The LAVSA was exposed to 15 different gases from 50 ppb to 5 ppm to monitor their performance (Fig. S12a–o). Each sensor had a different average performance value for each gas (Fig. 5e), which demonstrated the selective performance of the LAVSA. Seven experiments were then conducted for each of these gases at a concentration of 5 ppm, and the data obtained were analyzed with the help of PCA. Due to the apparent differences in the functional groups of these five types of odor molecules, it could be seen that each type of gas molecule was clustered in the corresponding region, showing a good clustering feature, indicating that the LAVSA was capable of classifying these five types of gas molecules with the help of PCA (Fig. 5f).

In addition, a new algorithm t-distributed stochastic neighborhood embedding (t-SNE) method was introduced to distinguish these 15 gas molecules [68]. After extracting the features from the sensor data and dimensionality reduction, a t-SNE plot (Fig. 5g) was drawn, with the values of the axes representing the differences in the features of different gases. As could be seen in the graph, the 15 gases were clustered separately and were clearly distinguishable. The results showed that with the assistance of the t-SNE algorithm and the PCA algorithm, the LAVSA had excellent sensitivity and selectivity, and could accurately identify both the five types of odor molecules and the 15 specific gases. In conclusion, the testing of the 15 odor molecules showed that the LAVSA consisting of BP and Ti_3_C_2_T_x_ had excellent sensitivity and selectivity, and had the capability for further practical applications.

### CHD Patient Identification on LAVAS-Based IGSP

**Fig. 6:**
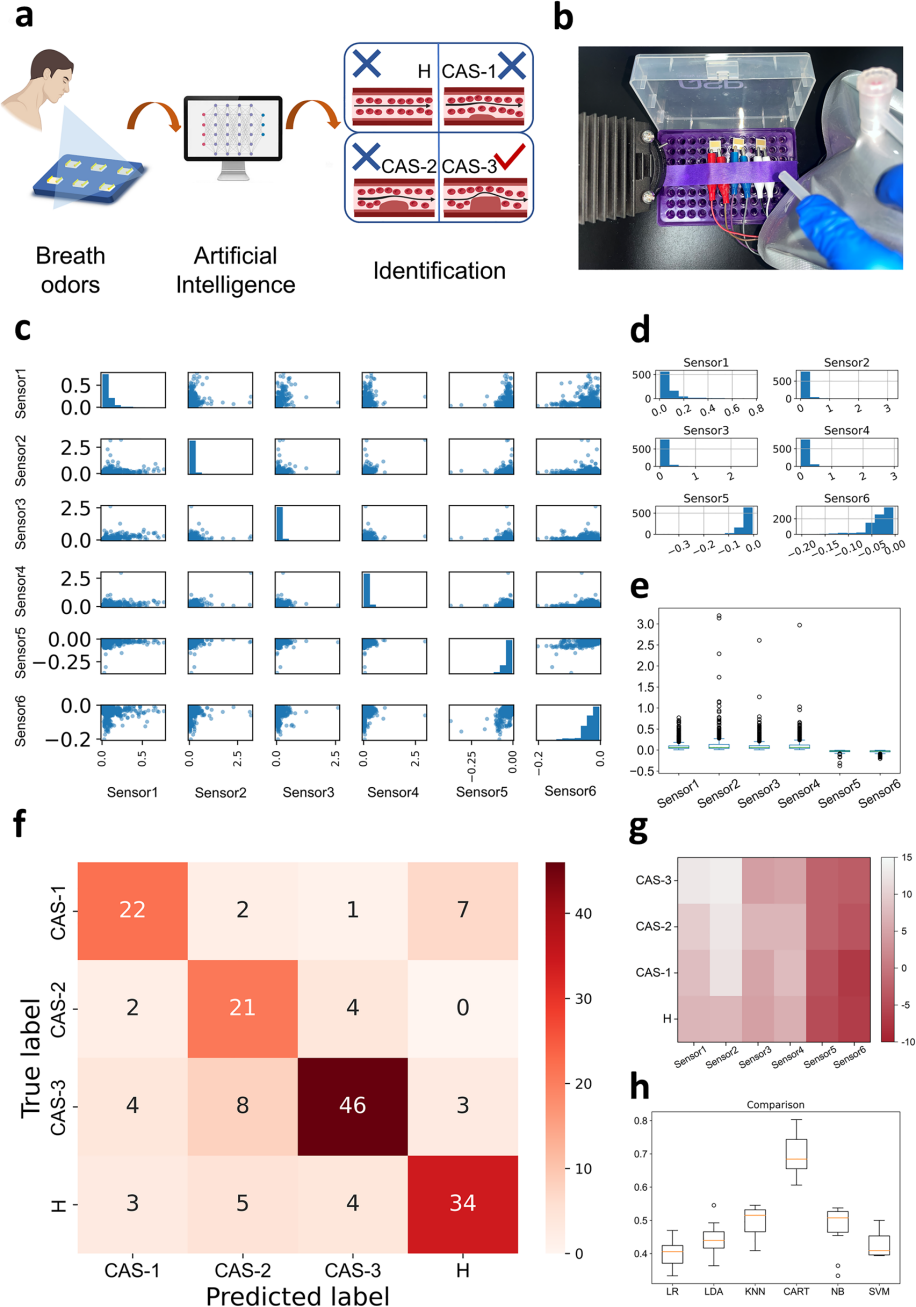
Illustration and outcome of CHD recognition via breath odors. **a** Diagram of the disease recognition with IGSP, **b** Real picture of the IGSP on testing breath odors. Statistical graphics of **c** Scatter matrix, **d** Histogram, and **e** Box plot of the performance of individual sensors in LAVSA to breath odors. **f** Results of the confusion matrix for recognizing four types of volunteers, including H, CAS-1, CAS-2, and CAS-3. **g** Average performance of the IGSP to breath odors. **h** Comparison of the accuracy of six types of ML algorithms recognizing different types of volunteers via breath odors. Figures were created with BioRender

Gas sensing had great potential for healthcare applications such as disease diagnosis. In this work, the detection and diagnosis of CHD patients by LAVSA-based IGSP with the help of an ML algorithm was explored (Fig. 6a). The diagnostic criteria for CHD was usually the degree of coronary artery stenosis (CAS), where 0–25% was considered mild, 25–50% was considered moderate, as well as greater than 50% was considered severe, and when the degree of CAS was greater than 50% it could be adjudged to be suffering from CHD [69, 70]. The rationale for non-invasive diagnosis through exhaled breath was that the body’s exhaled gas encompassed its unique metabolic profile. Therefore, the composition of exhaled gas in patients with varying degrees of CAS might differ from that of exhaled gas in healthy individuals. Multidimensional feature signals could be collected by IGSP and classified with the help of various mathematical algorithms. Since exhaled breath consisted of various components, depending on the biomarkers in it, each person had their own unique signals, and the LAVSA could detect these electrical signals and further differentiate them with the help of ML algorithms. The experiment was designed with a total of 45 participants (Table S3), including 10 healthy individuals (H), 11 patients with degree of CAS 0–25% (CAS-1), 9 patients with degree of CAS 25%–50% (CAS-2), and 15 patients with degree of CAS more than 50% (CAS-3).

The complexity of odor components in the exhaled gas and the differences in exhaled gas odor components between patients with different degrees of CAS and the healthy population was the basis for identification and classification. The samples from H, CAS-1, CAS-2, and CAS-3, were subjected to gas chromatography-mass spectrometry (GC–MS) to determine the differences in the odor components of exhaled gases in different populations, respectively. The GC–MS results (Fig. S14) visually showed that there were some differences in exhaled odors among different categories of people. Analyzed and further summarized, as shown in Tables S4–S7, the H samples contained components of ethyl acetate, acetone, and 2-ethyl-1-hexanol and so on. The CAS-1 samples contained components of 2,2,4-trimethyl-1,3-pentanediol diisobutyrate, acetone, ethyl acetate, etc. The CAS-2 samples contained components of 2-ethyl-1-hexanol, acetone, ethyl acetate, and so on. The CAS-3 samples contained components of acetone, ethyl acetate, ethanol, and so on. The diversity of compositions indicated that the biomarkers contained in the different types of samples were clearly recognizable.

In this work, the IGSP was utilized with the assistance of ML algorithms to process odor data exhaled by different volunteers and identify exhaled gases with unique sample categories. Samples were first collected via a sealed gas bag with a volume of 1 L, then the sample gas was placed 2 cm above the LAVSA (Fig. 6b), and the sample bag was gently pressed. The test was repeated 20 times for each sample. The data size was 45 people × 20 experiments, which meant 900 sets of data were obtained. In terms of the performance of the gas detection platform, the mean response (Fig. 6g) showed that the gas detection platform responded significantly to all four types of breath samples. In terms of the selectivity of the gas detection platform, the IGSP responded differently to each category of volunteer exhaled gas based on the various compositions of different categories of exhaled gas samples.

To detect and identify gas samples through the IGSP, ML algorithms were utilized to help achieve this. First, the data had to be pre-processed by organizing it into a size of 20 × 45 and further classified into four labels according to four categories. The data was then used for algorithm training and optimization. The histogram (Fig. 6d) and the boxplot diagram (Fig. 6e) showed the distribution of gas sensing performance of individual sensors in the LAVSA, showing the diversity of responses to various types of gas samples. The mathematical relationships between the sensors were shown in a scatter matrix plot (Fig. 6c), indicating significant differences between the signals. Six ML algorithms were used for data processing, namely logistic regression (LR), linear discriminant analysis (LDA), K-Nearest Neighbor algorithm (KNN), classification and regression tree (CART), Naive Bayes model (NB), and support vector machine (SVM) [33, 71–77]. From the confusion matrix (Fig. 6f), it could be seen that for the four categories of participants from different populations, H was predicted with 77% accuracy, CAS-1 predicted with 71% accuracy, CAS-2 predicted with 58% accuracy, and CAS-3 with 84% accuracy. Figure 6h showed the prediction results of the six algorithms through 10 cross-validation tests. CART had the best performance with an accuracy of 0.692, which meant that it performed well in recognizing breath odors. In comparison, LR had an accuracy of 0.403, which proved that it was not suitable for inter-sample identification. However, the IGSP had some limitations, such as it required known calibration samples for comparison to achieve accurate detection, which required an extensive library of calibration samples, and the detection of unrecorded gas categories remained an open issue. Also, determining the composition and corresponding concentration of exhaled gases through the IGSP remained challenging.

### IGSP Sensing Mechanism

**Fig. 7:**
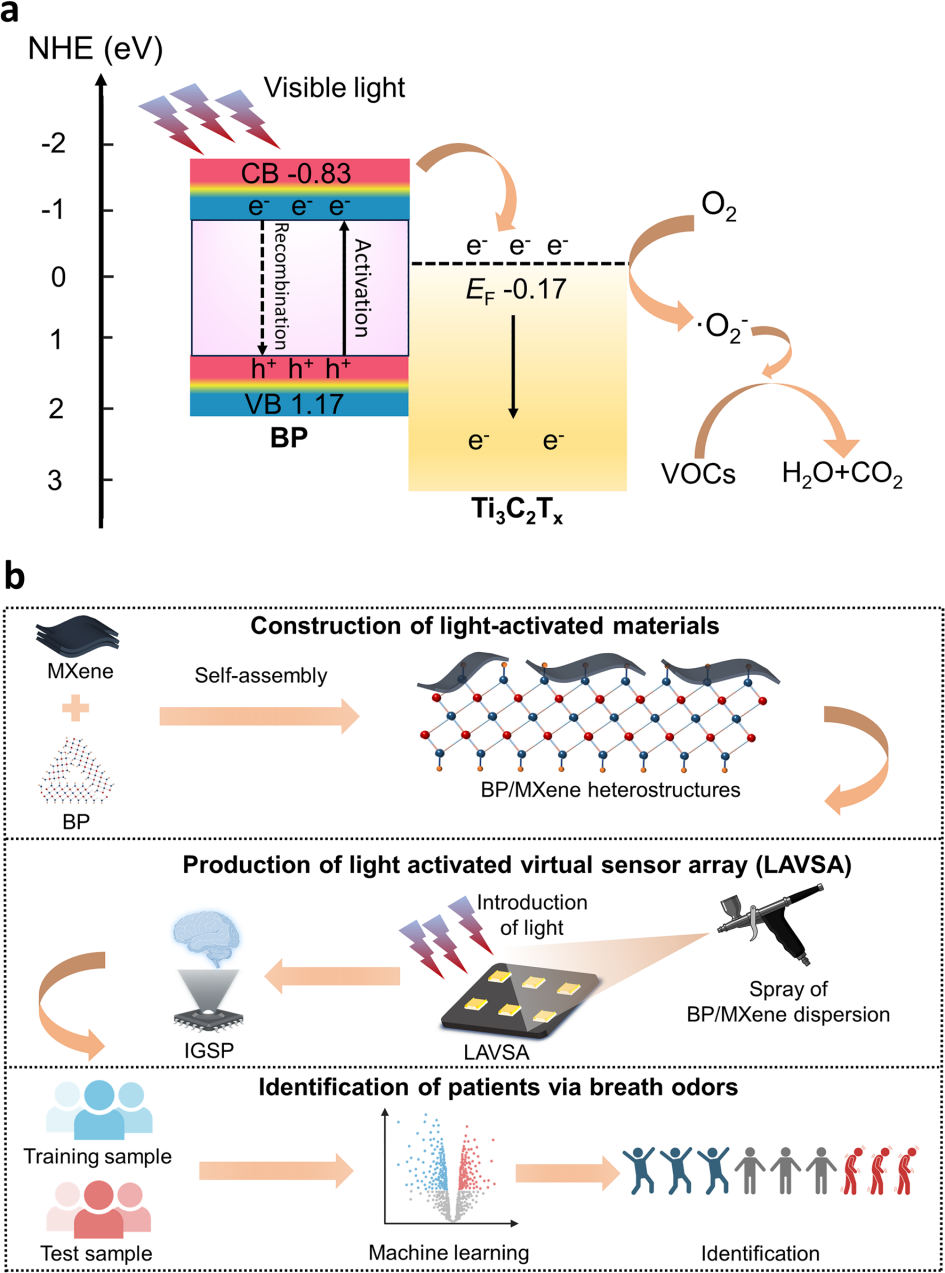
The mechanism of enhanced sensing property of the IGSP. **a** Change of conductivity pathway of the BP/Ti_3_C_2_T_x_ gas sensor after adsorption due to light activation. **b** Diagram of the improved sensing performance of the fabricated IGSP in disease recognition. Figures were created with BioRender

The gas-sensitizing properties of BP/Ti_3_C_2_T_x_ nanocomplexes were enhanced under visible light irradiation compared to pristine Ti_3_C_2_T_x_ (Fig. 7a). This was mainly due to the synergistic effect between BP and Ti_3_C_2_T_x_, as well as the increased electron exchange due to photon activation. On the one hand, the self-growth of BP nanosheets could lead to higher specific surface area and active sites, which resulted in enhanced gas adsorption and desorption processes. On the other hand, the heterostructure formed by BP and Ti_3_C_2_T_x_, as well as the modulation of carrier density through photon excitation, were important factors to improve the gas-sensitive performance of the sensor. According to the literature, the conduction band (CB) potential and valence band (VB) potential of BP were 0.83 and 1.17 eV, respectively [78]. Under visible light irradiation, photo-excited electrons were transferred from the VB to the CB of the BP. Because of the lower Fermi energy level (E_F_) equal to − 0.17 eV of Ti_3_C_2_T_x_, the electrons would be further transferred to Ti_3_C_2_T_x_ [79]. Since the E_F_ of Ti_3_C_2_T_x_ was more negative than the potential of O_2_/^·^O_2_^−^ equal to 0.046 eV and Ti_3_C_2_T_x_ had excellent conductivity (4600 ± 1100 S cm^−1^), the acquired photogenerated electrons were rapidly transferred to the surface of Ti_3_C_2_T_x_ and eventually captured by O_2_ [78, 80, 81]. Gas molecules such as VOCs absorbed ^·^O_2_^−^ electrons on the surface of Ti_3_C_2_T_x_ to generate CO_2_ and H_2_O. Therefore, the electron exchange between the target gas and the surface of the BP/Ti_3_C_2_T_x_ was enhanced through the above process. In all, the enhanced gas sensitivity performance of the BP/Ti_3_C_2_T_x_-based sensor was attributed to the formation of heterogeneous structure, the increase of active site, and photomodulated carrier density.

The improved gas sensing performance of the prepared IGSP could be attributed to three main factors (Fig. 7b). First, the construction of BP/Ti_3_C_2_T_x_ heterostructured complexes capable of photoresponsiveness provided a rich material base for high-performance gas sensing and facilitated the development of LAVSA. Second, the LAVSA consisted of three BP/Ti_3_C_2_T_x_ with different compositions, which operated under the presence and absence of light conditions, thus enabling the acquisition of multidimensional data to improve the characterization of the target gas and facilitating data collection and processing by combining with pattern recognition algorithms. Finally, with the help of ML algorithms, the data obtained by LAVSA could improve the differentiation of target gas characteristics. Comparative analysis of multiple ML algorithms allowed training on known samples, thus selecting the most accurate model for determining sample attribution. In conclusion, through improved material screening and preparation, data collection, and processing, the IGSP demonstrated excellent performance in gas detection and identification.

## Conclusions

In summary, the goal of the research was to establish an LAVSA and IGSP for the identification of different populations by human odor and for non-invasive diagnosis of CHD. In this work, from the perspective of photomodulation, BP was screened from materials whose light absorption range overlapped with that of MXene, and the BP/Ti_3_C_2_T_x_ complex was synthesized with MXene using a self-assembly strategy. As a proof-of-concept, sensors were fabricated using BP/Ti_3_C_2_T_x_ and tested against ammonia, acetone, ethanol, and ether gases to determine the optimal values of light wavelength and intensity as well as material ratios. Due to the synergistic effect of BP and MXene as well as photoexcitation, the complexes showed higher gas-sensitive performance, with a 26% higher response under light than in the absence of light, as well as an improvement in selectivity. On this basis, LAVSA was prepared using three different ratios of BP/Ti_3_C_2_T_x_ materials and tested for fifteen odor molecules under light and no light conditions, respectively. The recognition and classification of different gases were successfully realized with the help of PCA and t-SNE pattern recognition algorithms, which demonstrated its selectivity. Finally, the IGSP fabricated based on LAVSA and Arduino platform was put to practical use, and 45 volunteers were selected for testing, including healthy people and patients with three different degrees of CAS. With the help of the ML algorithm, the IGSP achieved an accuracy of 77%, 71%, 58%, and 84% in recognizing four different populations, H, CAS-1, CAS-2, and CAS-3, respectively, by human breath, and showed good recognition performance in both CAS patients and healthy people.

Inspired by physical modulation, the concept of LAVSA was proposed, and the BP/Ti_3_C_2_T_x_ complexes were fabricated, providing a new approach for the development of novel gas sensors with high sensitivity and selectivity. Besides, the IGSP fabricated in this study provided ideas for non-invasive diagnosis of CHD and also offered ideal prospects for immediate detection and identification of other diseases, such as tumors, as well as other applications in immediate gas sensing scenarios. Despite the good performance of the prepared IGSP, it still faces challenges in practical applications, which are what future research needs to focus on. On the one hand, the responsiveness of LAVSA still needs to be improved. On the other hand, the current training database was small in size and had limitations in large-scale applications. To settle these problems, there are three areas that can be addressed in future research. First, in terms of material and SA design, light-responsive materials could be further screened. Better-structured materials could be designed and synthesized to enhance the sensitivity. Besides, the wavelength and lightness of light and time could be further optimized to improve the accuracy and sensitivity. Second, regarding data collection, more data is needed to increase the stability of the IGSP. In future studies, gases from more scenarios will be detected, such as exhaled gas samples from lung cancer patients and stomach cancer patients. By collecting more data, a large database will be constructed for more applications. Finally, regarding data processing, more accurate ML algorithms need to be designed. The machine learning algorithms used in the current study leave much to be desired in terms of accuracy. In future research, on the one hand, the accuracy of the ML algorithm model is trained by collecting more data. On the other hand, it is to enhance the discriminative ability by improving the ML algorithm, such as adopting neural networks.

## Supplementary Information

Below is the link to the electronic supplementary material.

Supplementary file1 (DOCX 8426 KB)
