# Supplementary material for: Light-Activated Virtual Sensor Array with Machine Learning for Non-Invasive Diagnosis of Coronary Heart Disease
Source: Nanomicro Lett. 2024 Aug 16;16:274. doi: 10.1007/s40820-024-01481-7 (PMC11327237; doi:10.1007/s40820-024-01481-7)
Supplement: Supplementary file 1 — Supplementary file1 (DOCX 8426 KB) [file 40820_2024_1481_MOESM1_ESM.docx]

Supporting Information for

**Light-Activated Virtual Sensor Array with Machine Learning for Non-Invasive Diagnosis of Coronary Heart Disease**

Jiawang Hu^1,2,#^, Hao Qian^3,4,#^, Sanyang Han^5^, Ping Zhang^4,^*, Yuan Lu^1,2,^*

^1^ Department of Chemical Engineering, Tsinghua University, Beijing 100084, P. R. China

^2^ Key Laboratory of Industrial Biocatalysis, Ministry of Education, Tsinghua University, Beijing 100084, P. R. China

^3^ Department of Cardiology, Xuanwu Hospital, Capital Medical University, Beijing 100053, P. R. China

^4^ Department of Cardiology, Beijing Tsinghua Changgung Hospital, School of Clinical Medicine, Tsinghua University, Beijing 102218, P. R. China

^5^ Institute of Biopharmaceutical and Health Engineering, Shenzhen International Graduate School, Tsinghua University, Shenzhen 518055, P. R. China

# Jiawang Hu and Hao Qian contributed equally to this work.

*Corresponding authors. E-mail: [zpa00593@btch.edu.cn](mailto:zpa00593@btch.edu.cn) (Ping Zhang); [yuanlu@tsinghua.edu.cn](mailto:yuanlu@tsinghua.edu.cn) (Yuan Lu)

**Supplementary Figures and Tables**


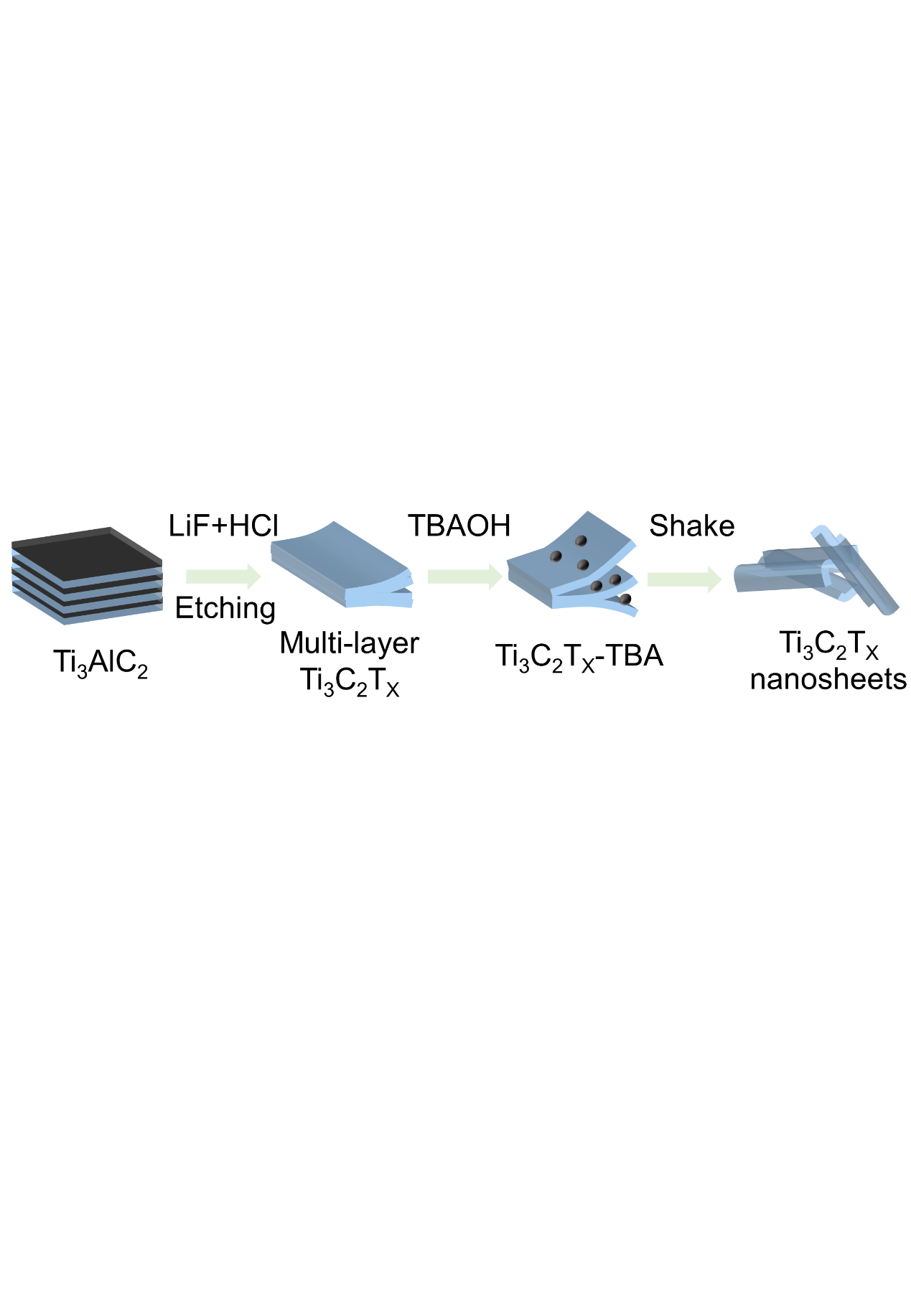


**Fig. S1** Schematic illustration of the fabrication of Ti_3_C_2_T_x_ nanosheets


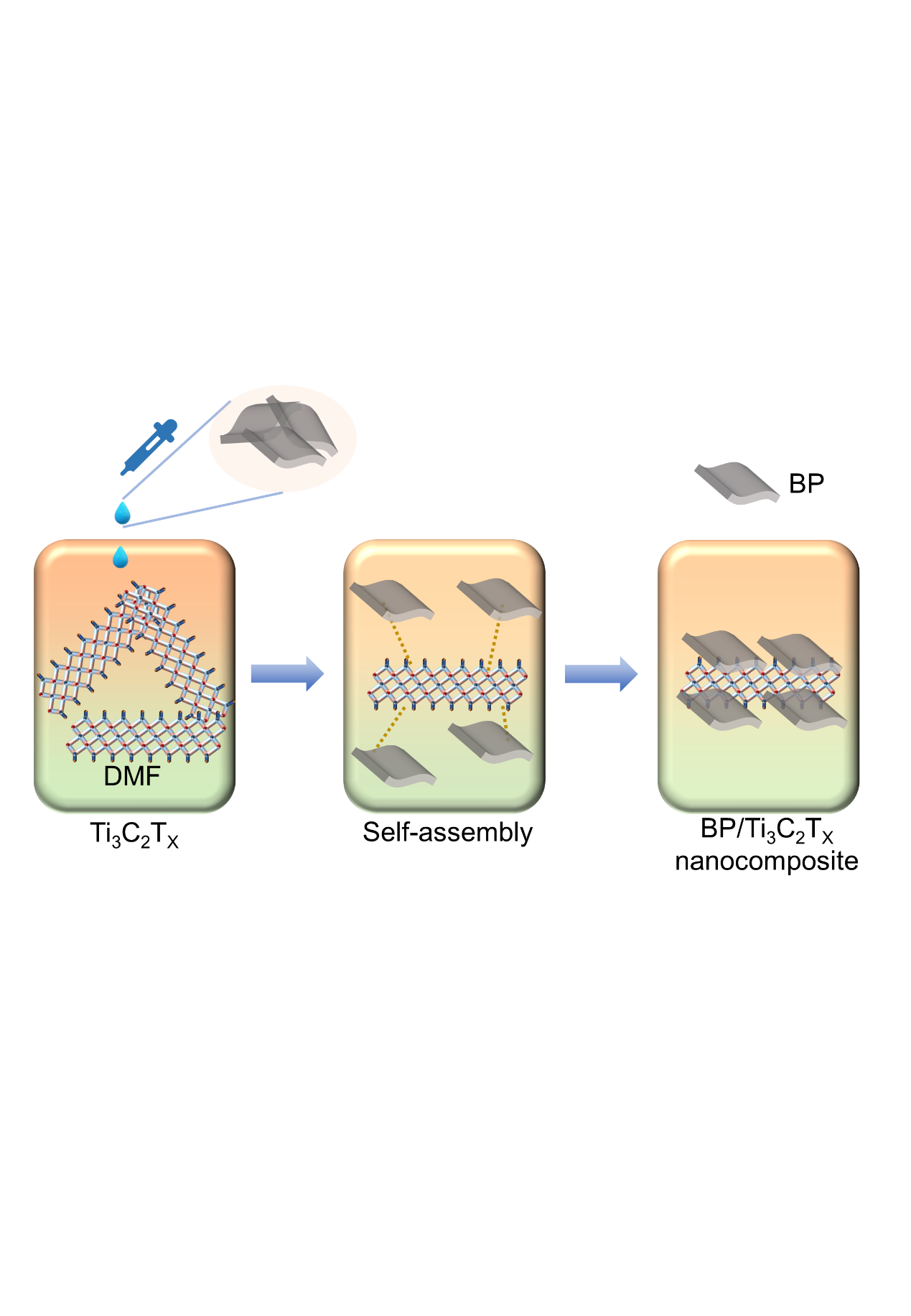


**Fig. S2** Schematic illustration of the fabrication of BP/Ti_3_C_2_T_x_ heterojunctions


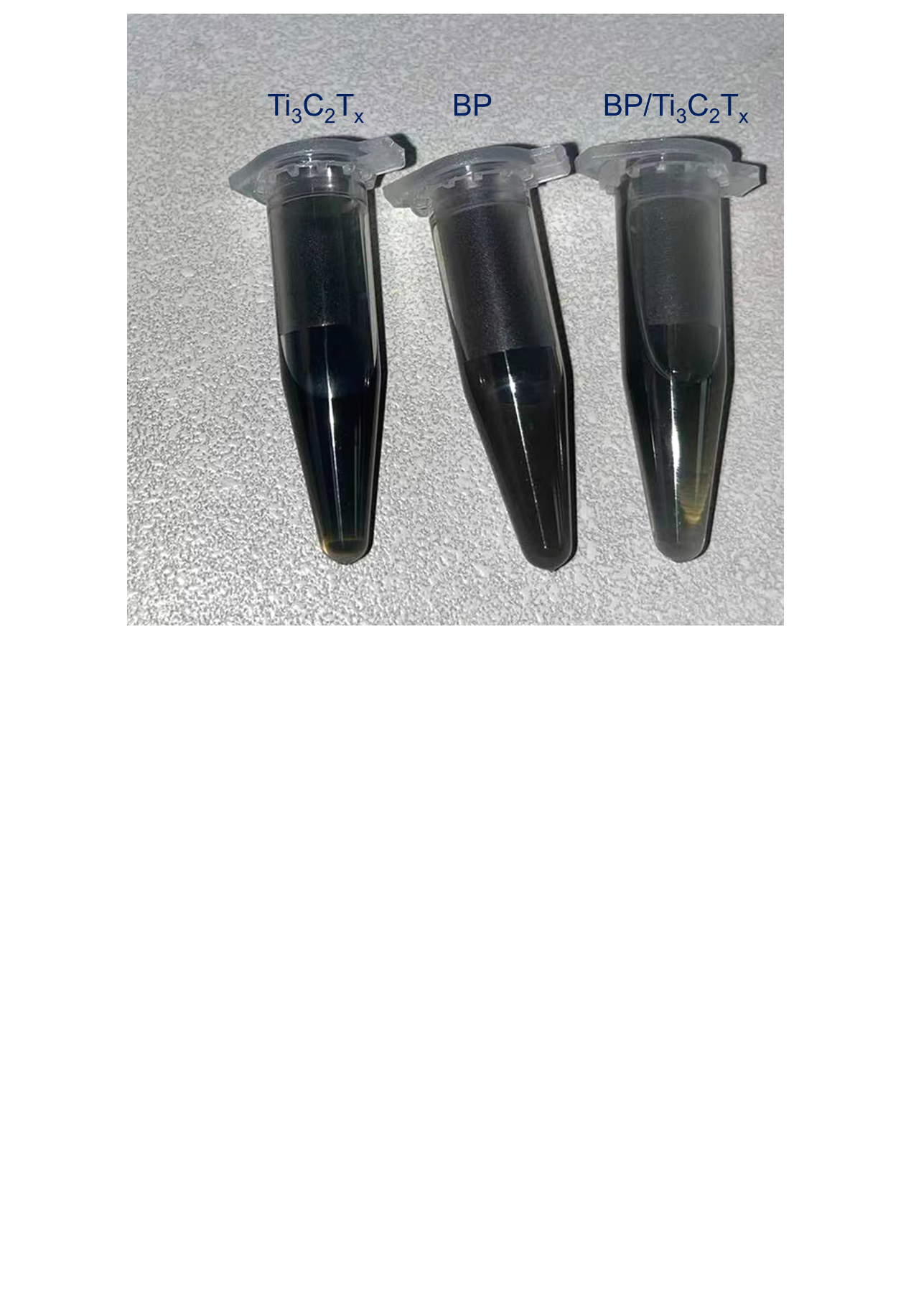


**Fig. S3** Image of the Ti_3_C_2_T_x_, BP and BP/Ti_3_C_2_T_x_ solution


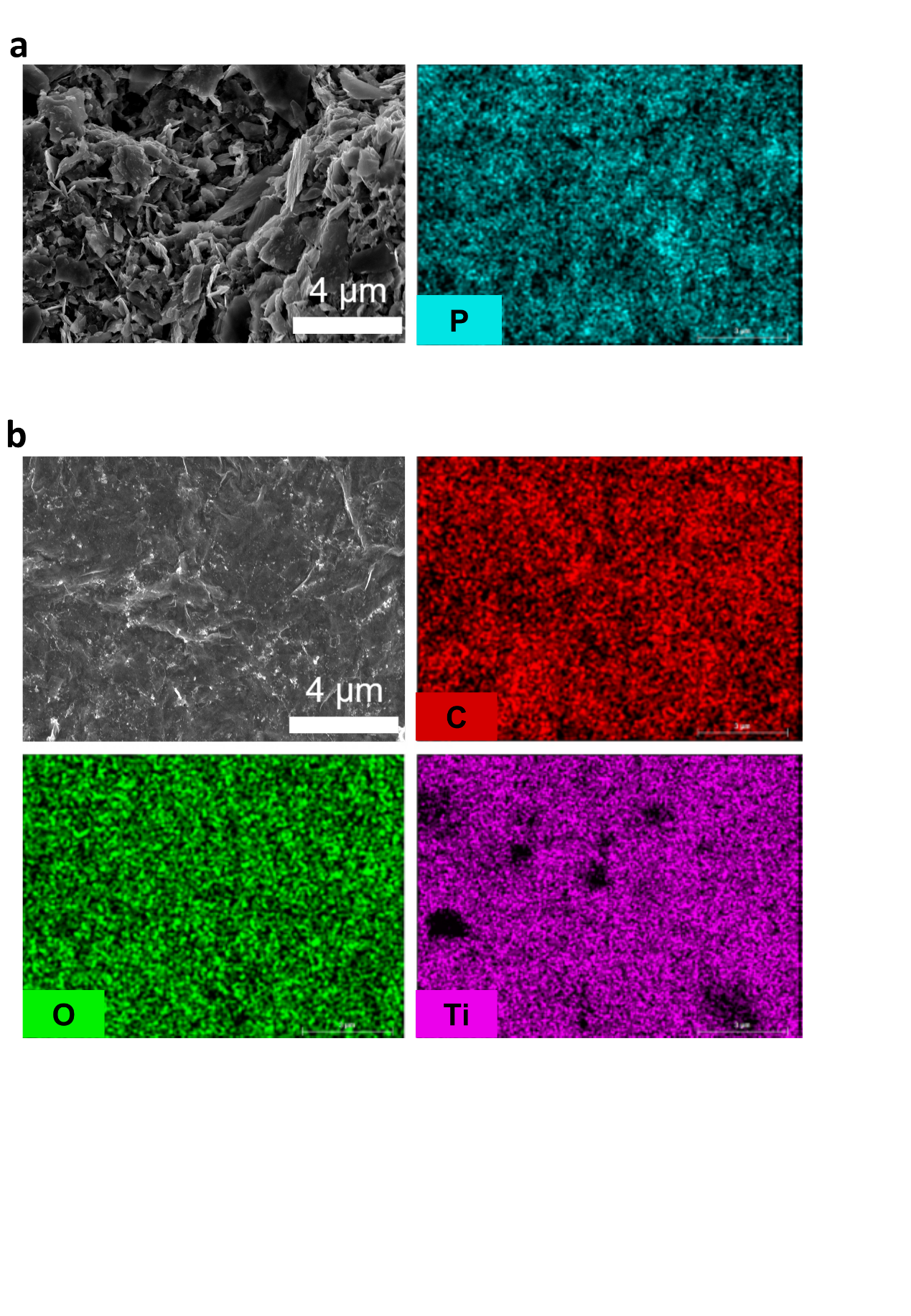


**Fig. S4** **a** SEM images of the BP nanosheets and **b** corresponding element mapping images of P. **c** SEM images of the Ti_3_C_2_T_x_ nanosheets and **d** corresponding element mapping images of C, O, and Ti


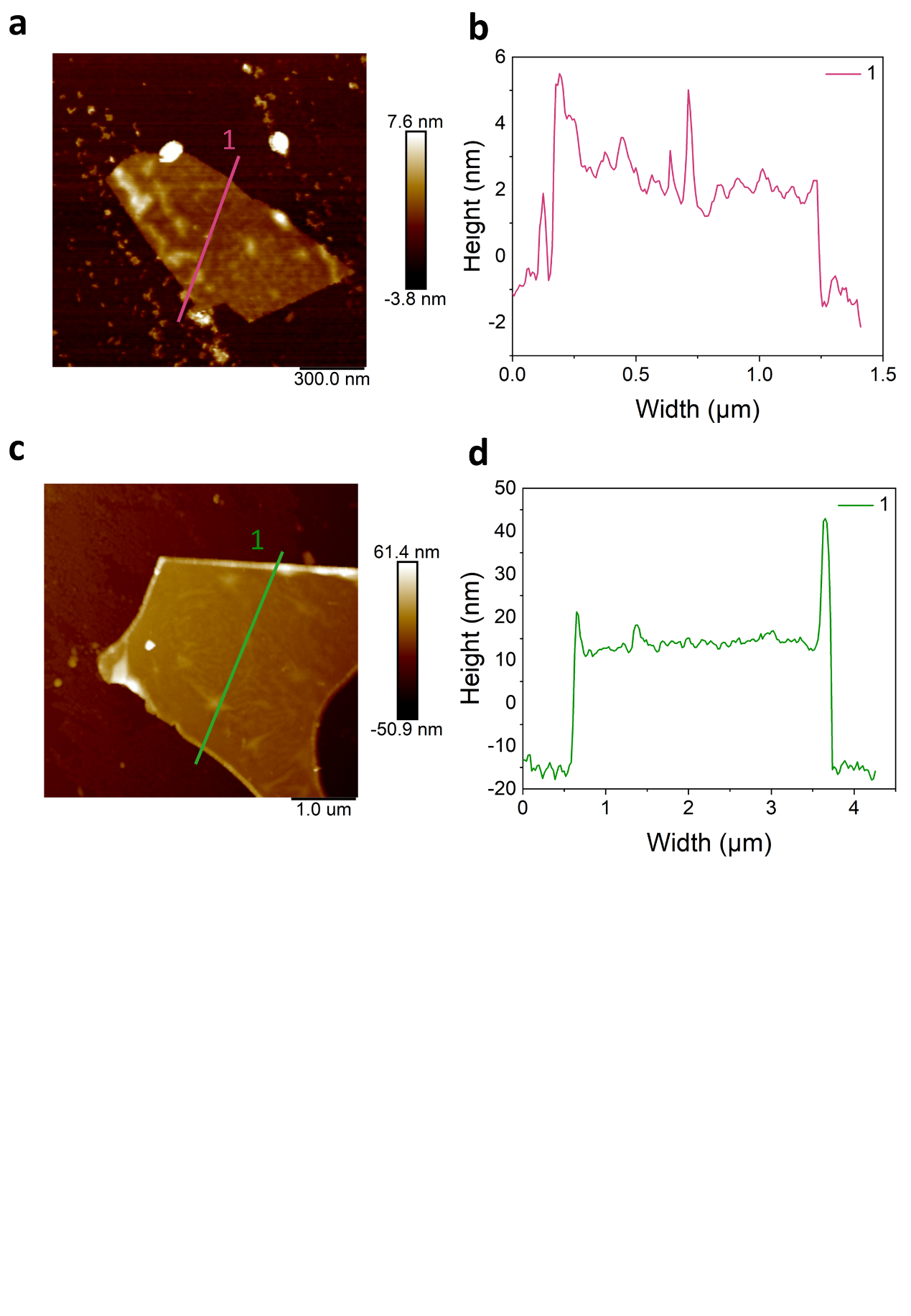


**Fig. S5** **a** AFM images of the Ti_3_C_2_T_x_ nanosheets and **b** corresponding height profile. **a** AFM images of the BP nanosheets and **b** corresponding height profile


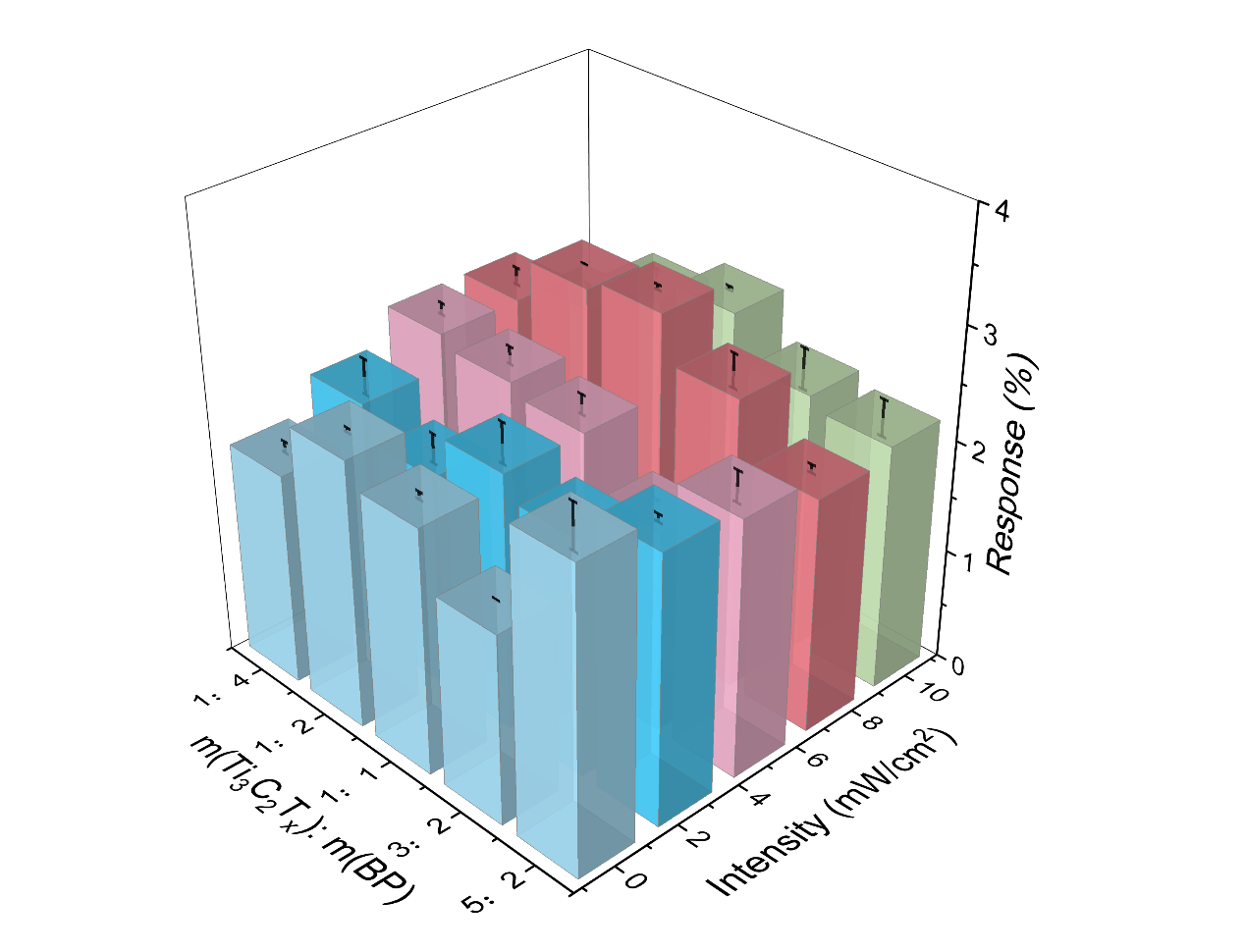


**Fig. S6** Response of different ratios of BP/Ti_3_C_2_T_x_ composite at different light intensities at 800 nm to acetone at the concentration of 5 ppm


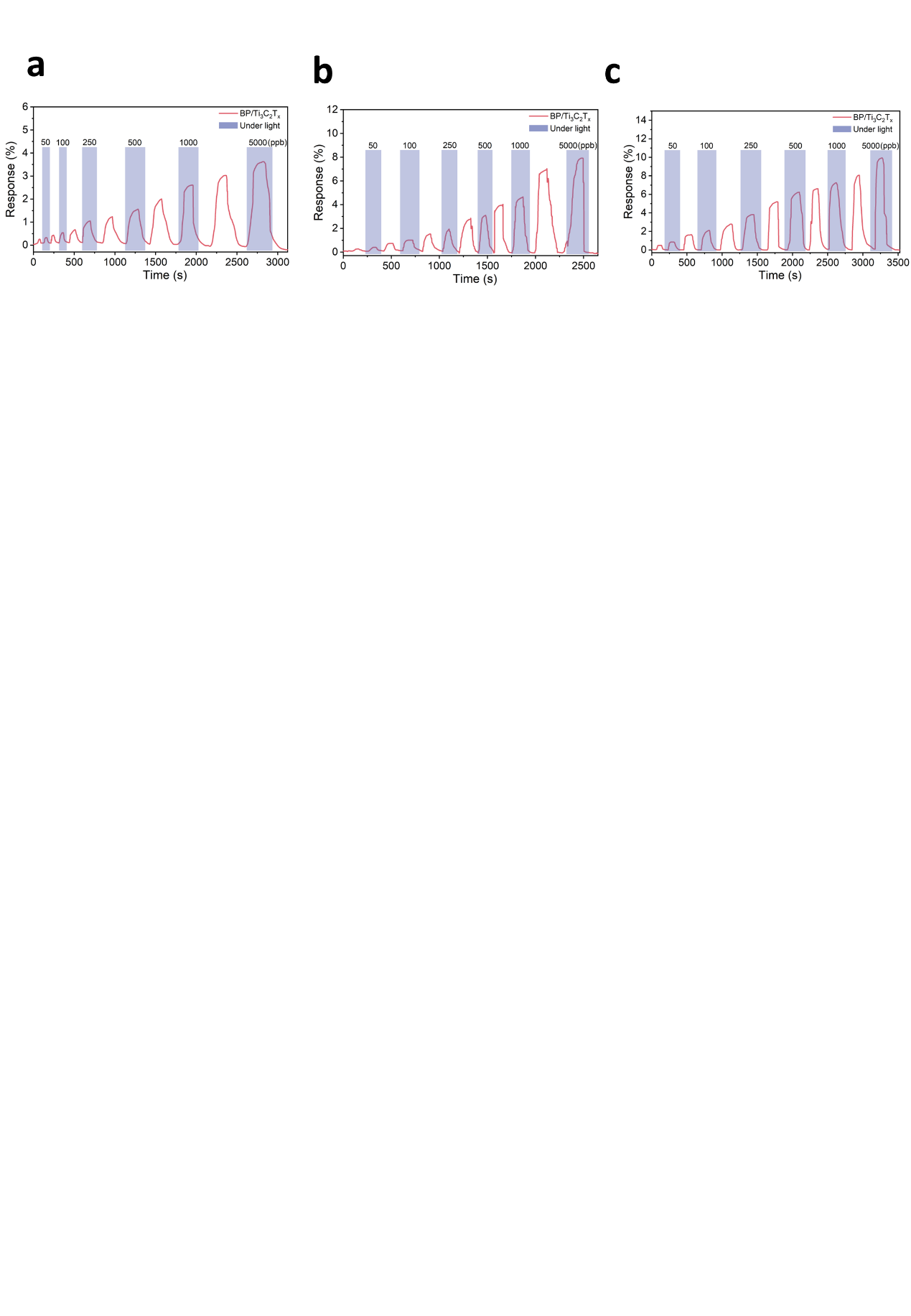


**Fig. S7** Comparison of real-time sensing response of gas sensors based on pristine BP/Ti_3_C_2_T_x_ composite to **a** ammonia, **b** ethanol, and **c** ether at concentrations ranging from 50 ppb to 5 ppm in the presence and absence of light


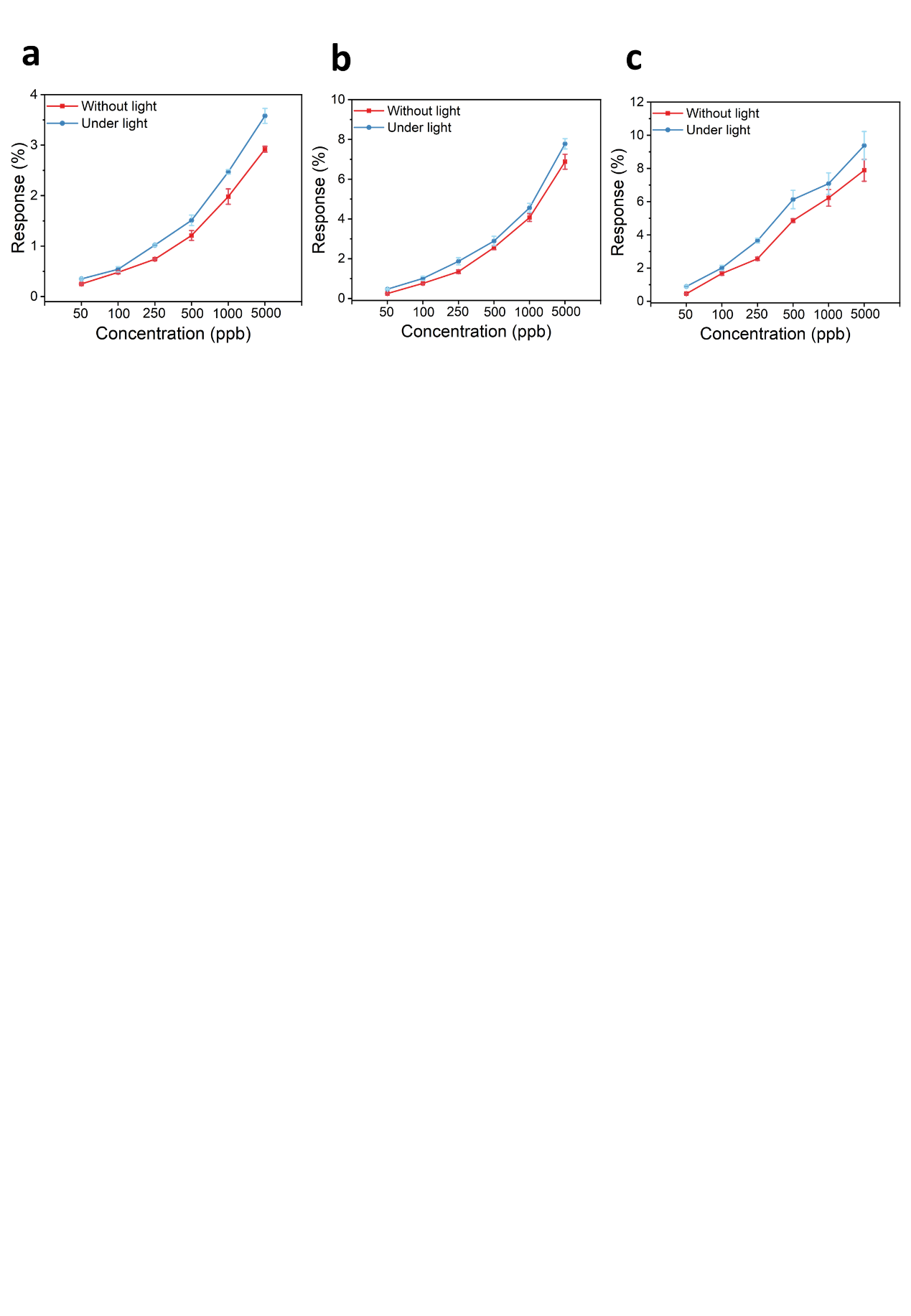


**Fig. S8** Comparison of the gas-sensitive performance of gas sensors based on BP/Ti_3_C_2_T_x_ composite to **a** ammonia, **b** ethanol, and **c** ether at the concentration ranging from 50 ppb to 5 ppm in the presence and absence of light


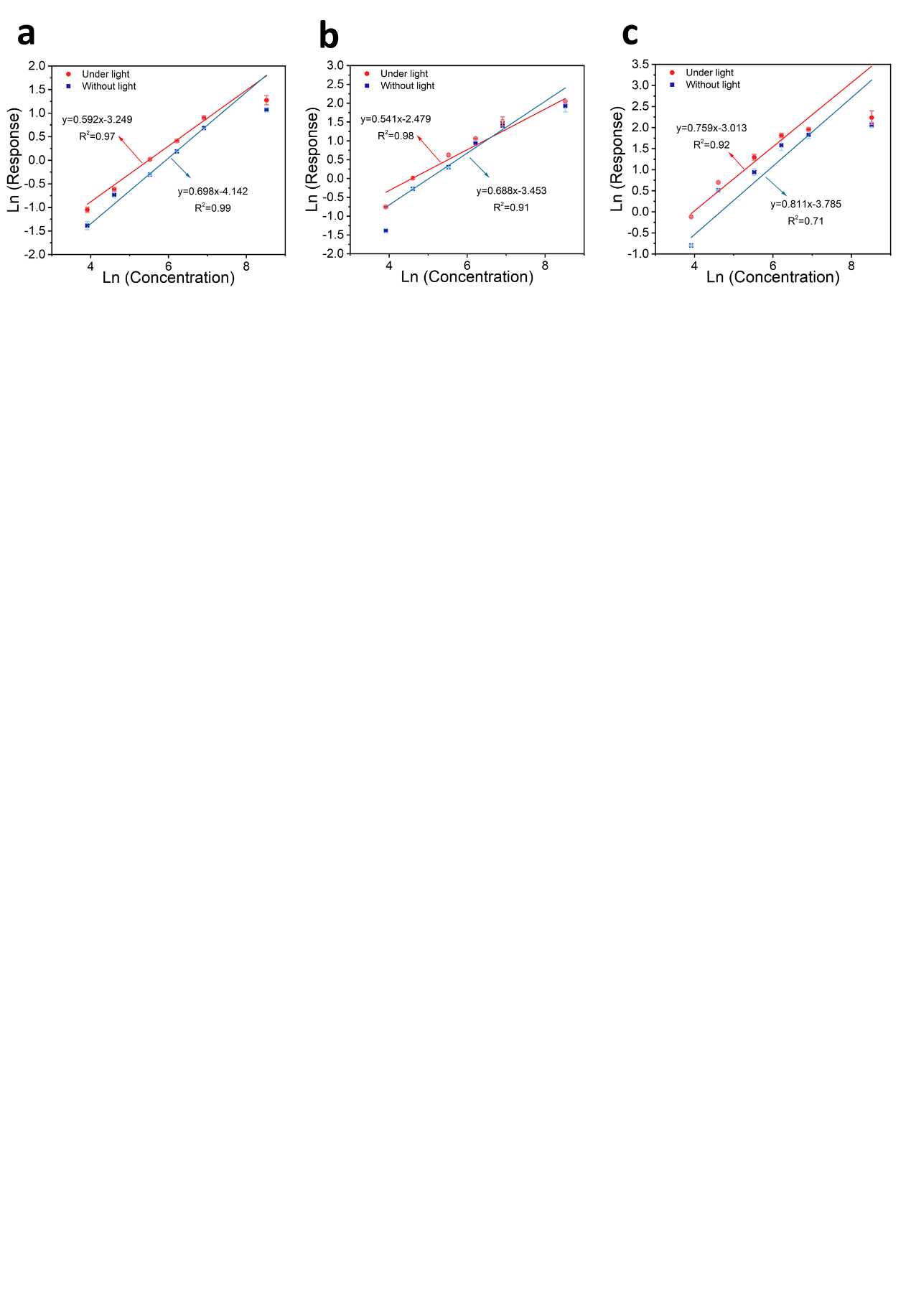


**Fig. S9.** Comparison of the linear relationship between the logarithm of the response and the logarithm of the gas concentration based on BP/Ti_3_C_2_T_x_ composite to **a** ammonia, **b** ethanol, and **c** ether in the presence and absence of light


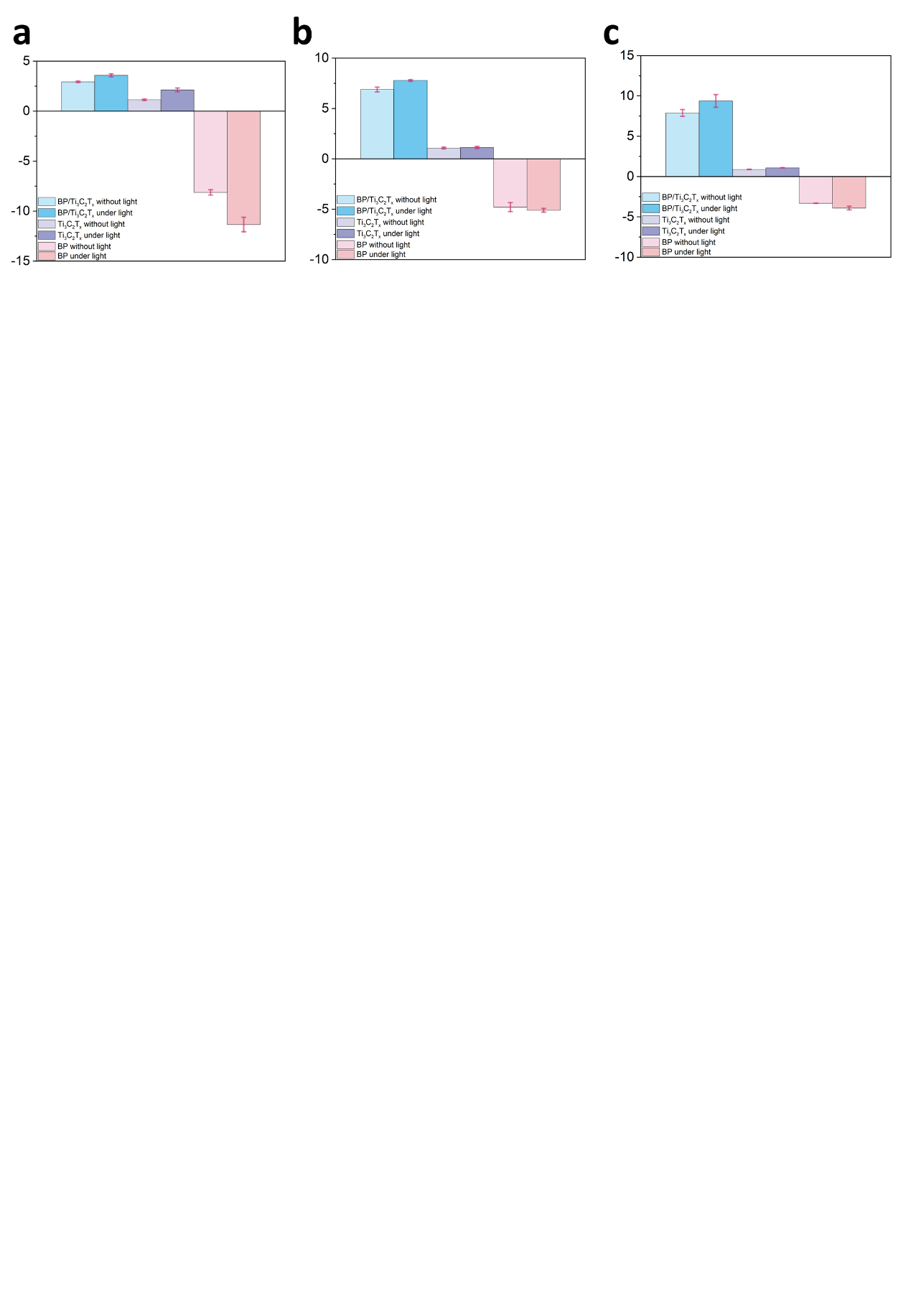


**Fig. S10** Comparison of the response of the Ti_3_C_2_T_x_, BP and BP/Ti_3_C_2_T_x_ composite to **a** ammonia, **b** ethanol and **c** ether at the concentration of 5 ppm in the presence and absence of light


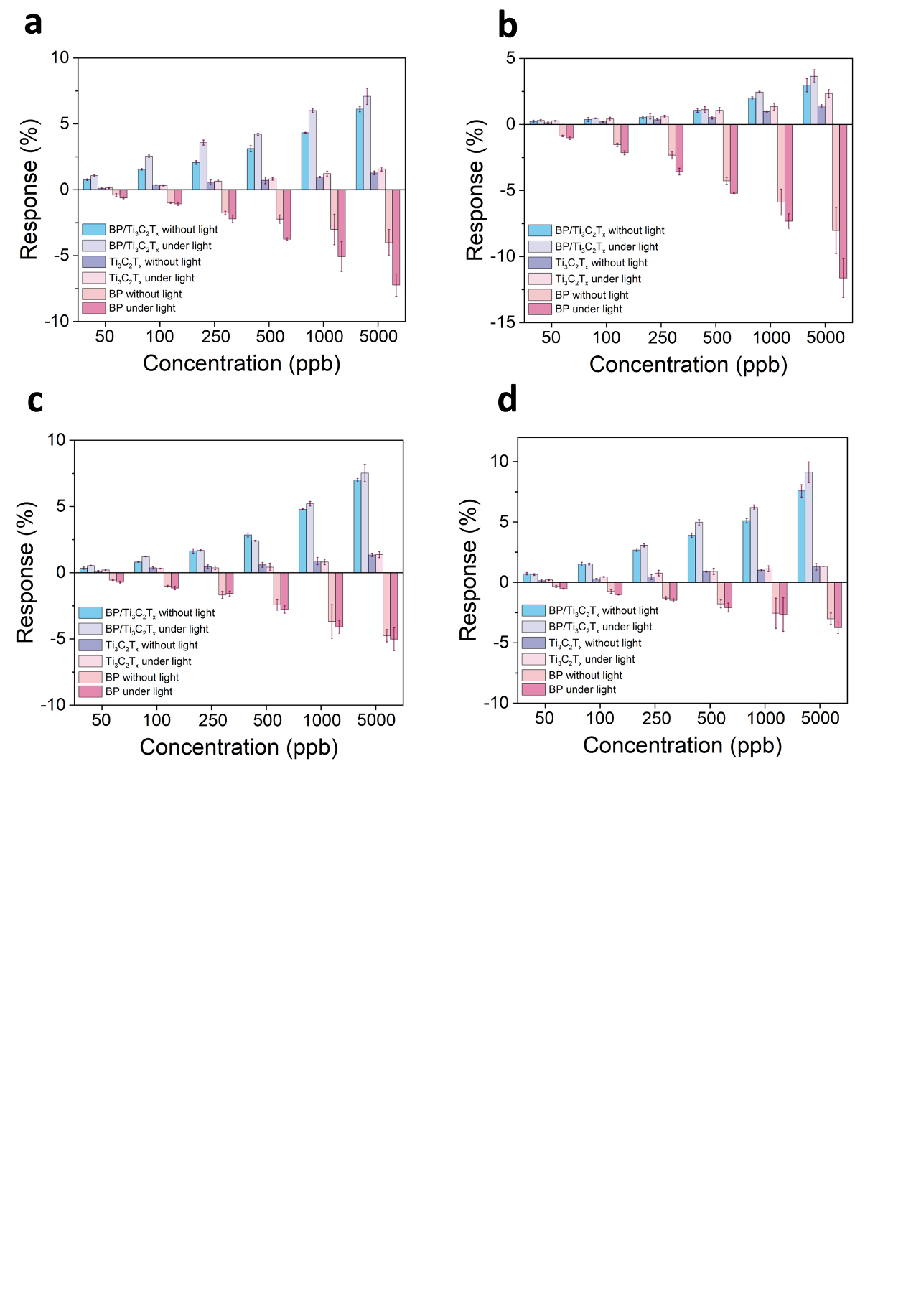


**Fig. S11** Response of LAVSA to **a** acetone, **b** ammonia, **c** ethanol, and **d** ether at the concentration ranging from 50 ppb to 5 ppm


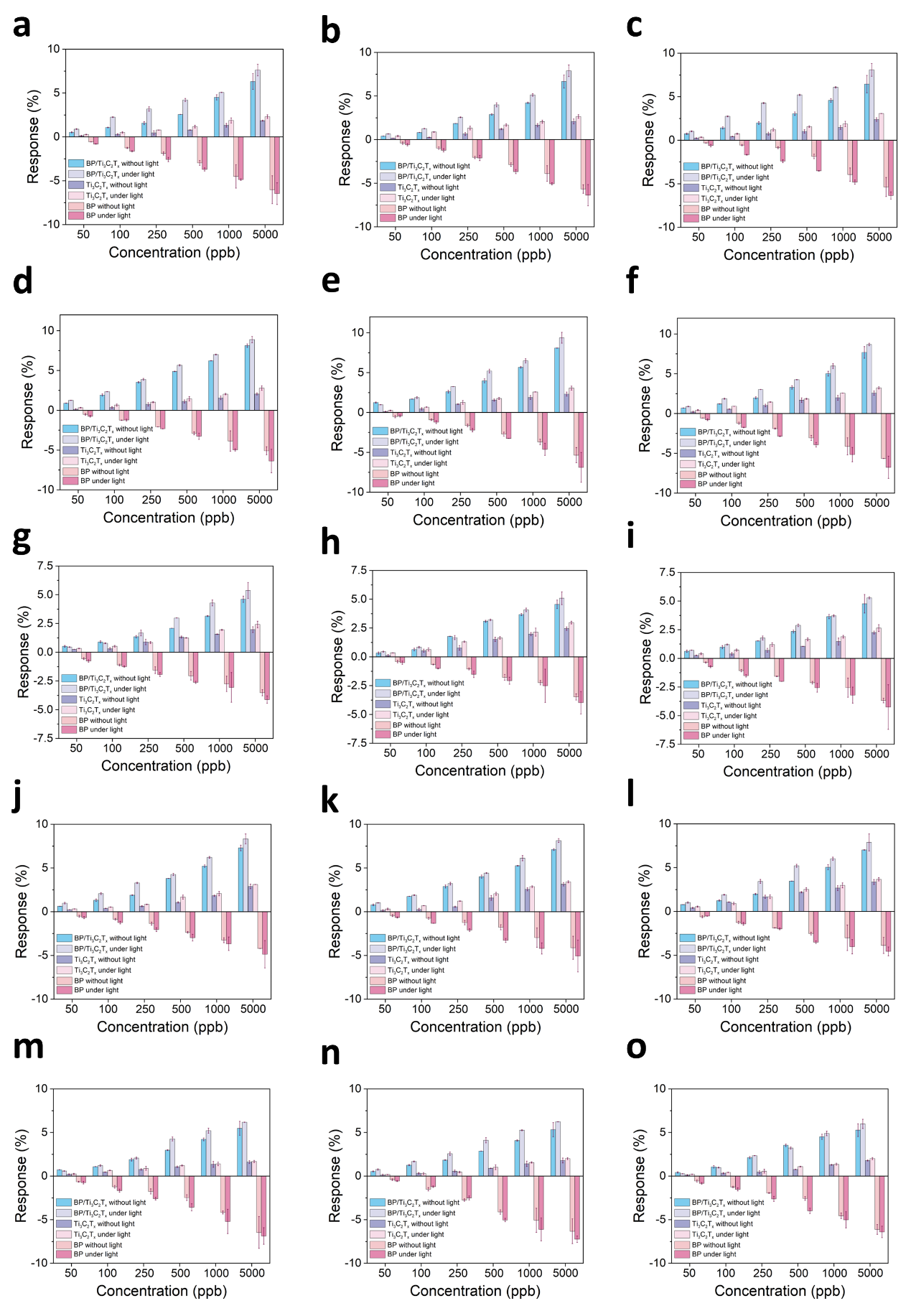


**Fig. S12** Response of LAVSA to fifteen odor molecules: **a** n-Octanol, **b** 1-Nonanol, **c** Citral, and **d** Safranal, **e** Nonanal, **f** Ethyl isobutyrate, **g** Prenyl acetate, h) γ-Caprolactone, **i** 2-Octanone, **j** 2-Heptanone, **k** S-(+)-Carvone, **l** Nonoic acid, **m** Heptanol, **n** Heptanoic acid, **o** n-Octanoic acid at the concentration ranging from 50ppb to 5ppm


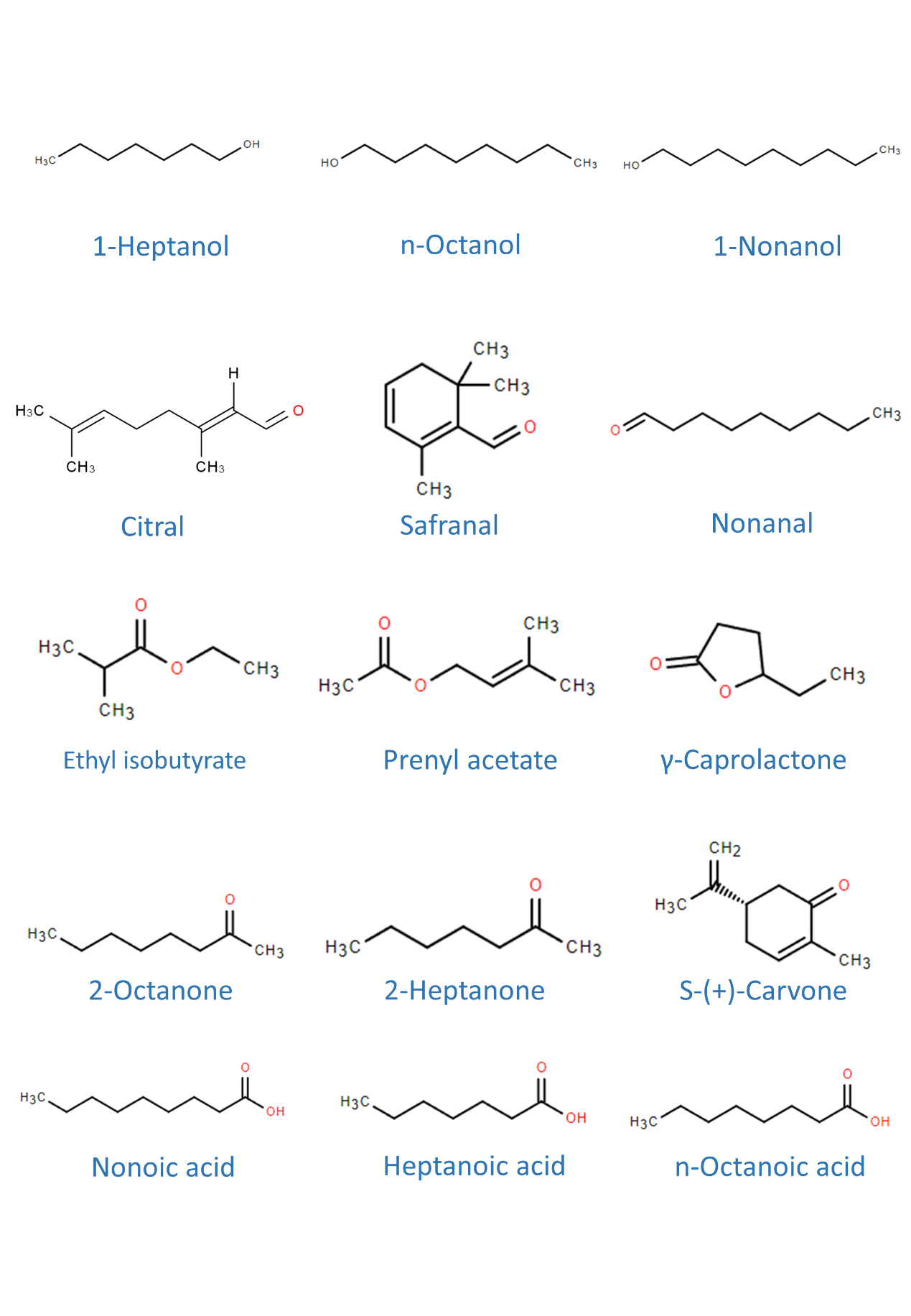


**Fig.** **S13** Chemical structures of fifteen odor molecules


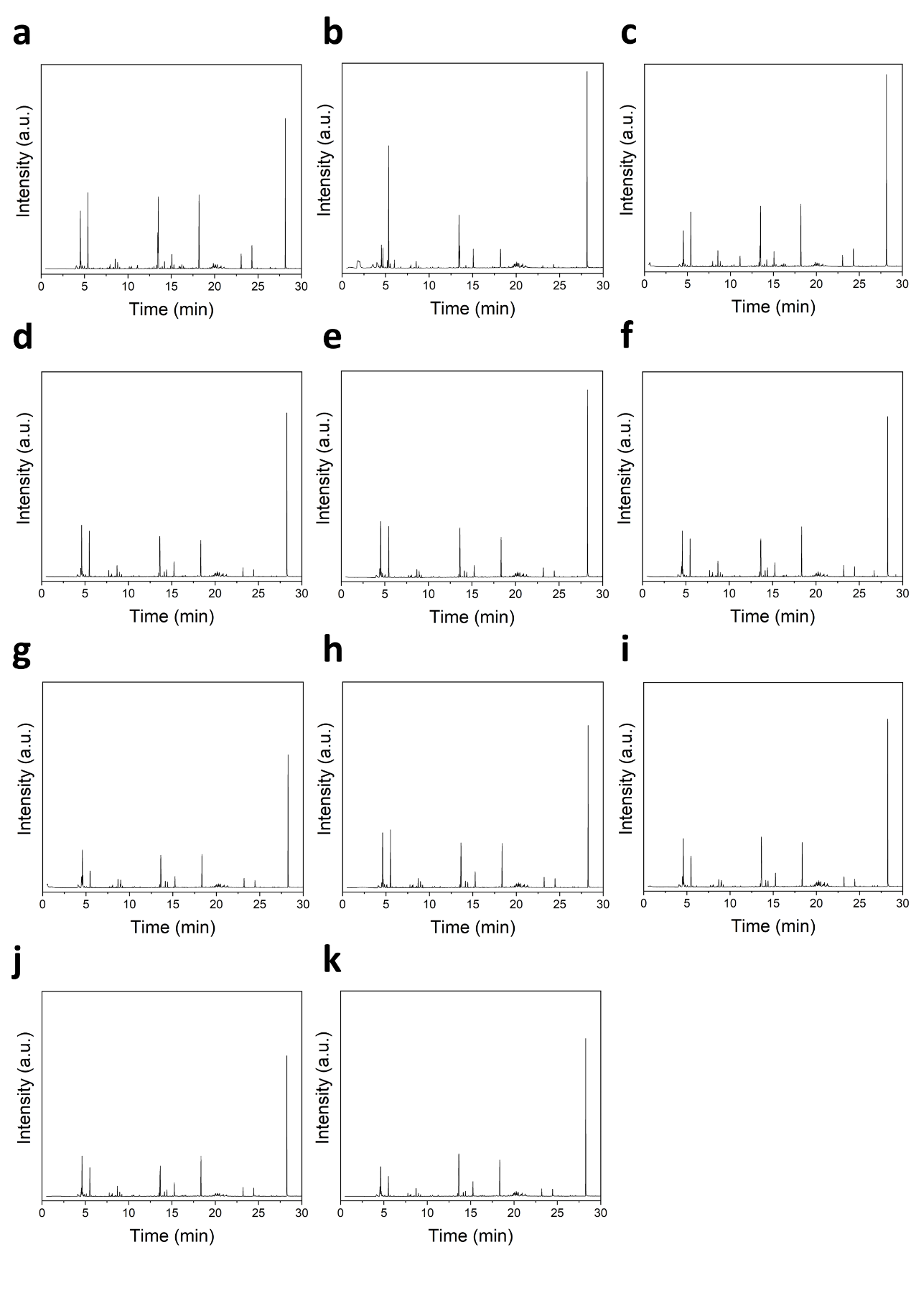


**Fig. S14** Gas chromatography (G**C** of different odor samples. GC of **a** HJW, **b** HLW, and **c** QCT affiliated with H population. GC of **d** XJF, **e** JBC, and **f** MQF affiliated with the CAS-1 population. GC of **g** HB, **h** DJX, and **i** XJ affiliated with the CAS-2 population. GC of **j** LK and **k** CRJ, and **l** WLT affiliated with the CAS-3 population

**Table S1**Comparison of gas-sensing performance of BP/Ti_3_C_2_T_x_ composite with other light activated sensors

| Sensitive Materials | Targeted gas | Conc.(ppm) | Light  source | Response | Refs. |
| --- | --- | --- | --- | --- | --- |
| CuO/MoS_2_ | NO_2_ | 10 | IR | 8.98 | [1] |
| SnS_2_ | NO_2_ | 8 | VIS | 12.5 | [2] |
| CuPc/ZnO | Ammonia | 100 | VIS | 15.8 | [3] |
| Bi_2_S_3_/SnS_2_ | NO_2_ | 0.5 | VIS | 14 | [4] |
| WS_2_ /PbS | NO_2_ | 0.02 | UV | 0.998 | [5] |
| NiS/Ni-ZnO | Formaldehyde | 10 | UV | 3.3 | [6] |
| ZnO/g-C_3_N_4_ | NO_2_ | 7 | VIS | 44.8 | [7] |
| Ag-ZnO | NO_2_ | 5 | VIS | 2.5 | [8] |
| g-C_3_N_4_/GaN | NO_2_ | 5 | UV | 7.8% | [9] |
| BP/Ti_3_C_2_T_x_ | Acetone | 5 | VIS | 7.21% | **This work** |

**Table S2.** Information of odor molecules

| Notation | Odor molecules | CAS Number | Manufacturers | Purity |
| --- | --- | --- | --- | --- |
| Alcohol-1 | 1-Heptanol | 111-70-6 | Macklin | AR |
| Alcohol-2 | n-Octanol | 111-87-5 | Macklin | AR |
| Alcohol-3 | 1-Nonanol | 143-08-8 | Macklin | AR |
| Aldehyde-1 | Citral | 5392-40-5 | Aladdin | AR |
| Aldehyde-2 | Safranal | 116-26-7 | Aladdin | AR |
| Aldehyde-3 | Nonanal | 124-19-6 | Aladdin | AR |
| Ester-1 | Ethyl isobutyrate | 97-62-1 | Macklin | AR |
| Ester-2 | Prenyl acetate | 1191-16-8 | Macklin | AR |
| Ester-3 | γ-Caprolactone | 695-06-7 | Aladdin | AR |
| Ketone-1 | 2-Octanone | 111-13-7 | Aladdin | AR |
| Ketone-2 | 2-Heptanone | 110-43-0 | Macklin | AR |
| Ketone-3 | S-(+)-Carvone | 2244-16-8 | Macklin | AR |
| Acid-1 | Nonoic acid | 112-05-0 | Macklin | AR |
| Acid-2 | Heptanoic acid | 111-14-8 | Aladdin | AR |
| Acid-3 | n-Octanoic acid | 124-07-2 | Aladdin | AR |

**Table S3** Information of volunteers

| Notation | Affiliated groups |
| --- | --- |
| HJW | H |
| HLW | H |
| LWC | H |
| QYD | H |
| QCT | H |
| WT | H |
| WXX | H |
| XXJ | H |
| YJZ | H |
| ZYL | H |
| YYS | CAS-1 |
| ZZH | CAS-1 |
| NGY | CAS-1 |
| WDJ | CAS-1 |
| DJX | CAS-1 |
| XJF | CAS-1 |
| JBC | CAS-1 |
| GQ | CAS-1 |
| WTL | CAS-1 |
| LSQ | CAS-1 |
| DMQ | CAS-1 |
| YRY | CAS-2 |
| HQ | CAS-2 |
| BXJ | CAS-2 |
| ZHW | CAS-2 |
| ZHB | CAS-2 |
| MQF | CAS-2 |
| HB | CAS-2 |
| XJ | CAS-2 |
| LW | CAS-2 |
| LSH | CAS-3 |
| LCZ | CAS-3 |
| LK | CAS-3 |
| CRJ | CAS-3 |
| LXX | CAS-3 |
| XHX | CAS-3 |
| SYZ | CAS-3 |
| GH | CAS-3 |
| SXM | CAS-3 |
| ZXF | CAS-3 |
| LRH | CAS-3 |
| LGL | CAS-3 |
| LGY | CAS-3 |
| ZRQ | CAS-3 |
| ZSP | CAS-3 |

**Table S4** Partial components of odor samples of H populations

| VOCs | Relative Concentration |
| --- | --- |
| Ethyl Acetate | 9.74 |
| Acetone | 9.6 |
| 2-Ethyl-1-hexanol | 2.05 |
| Octane | 1.28 |
| Decane | 1.03 |
| Formic acid | 0.87 |
| Nonane | 0.86 |
| Acetic acid | 0.72 |
| 1-Undecene | 0.62 |
| Acetaldehyde | 0.39 |

**Table S5** Partial components of odor samples of CAS-1 populations

| VOCs | Relative Concentration |
| --- | --- |
| 2,2,4-Trimethyl-1,3-pentanediol diisobutyrate | 22.66 |
| Acetone | 9.17 |
| Ethyl Acetate | 7.14 |
| 2-Ethyl-1-hexanol | 2.78 |
| Ethanol | 2.6 |
| Octane | 1.77 |
| 1-Nonanol | 1.58 |
| Undecane | 1.31 |
| 1-Heptanol | 1.3 |
| Isoprene | 1.07 |

**Table S6** Partial components of odor samples of CAS-2 populations

| VOCs | Relative Concentration |
| --- | --- |
| 2-Ethyl-1-hexanol | 12.82 |
| Acetone | 9.7 |
| Ethyl Acetate | 7.74 |
| Ethanol | 2.34 |
| 2-Ethyl-1-hexanol | 2.22 |
| 1-Nonanol | 1.63 |
| Carbamic acid | 1.33 |
| 1-Heptanol | 1.31 |
| Formic acid | 1.29 |
| Heptane | 1.22 |

**Table S7** Partial components of odor samples of CAS-3 populations

| VOCs | Relative Concentration |
| --- | --- |
| Acetone | 21.1 |
| Ethyl Acetate | 8.44 |
| Ethanol | 5.77 |
| 2-Ethyl-1-hexanol | 2.93 |
| Octane | 2.35 |
| 2,5-Dimethylhexane-2,5-dihydroperoxide | 1.84 |
| Undecane | 1.74 |
| Isoprene | 1.52 |
| 2-Isopropyl-5-methyl-1-heptanol | 1.33 |
| sec-Butyl acetate | 1.14 |
